# Comparison of Depression and Anxiety Following Self-reported COVID-19–Like Symptoms vs SARS-CoV-2 Seropositivity in France

**DOI:** 10.1001/jamanetworkopen.2023.12892

**Published:** 2023-05-11

**Authors:** Alexandra Rouquette, Arthur Descarpentry, Fallou Dione, Bruno Falissard, Stéphane Legleye, Cécile Vuillermoz, Anne Pastorello, Laurence Meyer, Josiane Warszawski, Camille Davisse-Paturet, Maria Melchior

**Affiliations:** 1 Centre de Recherche en Epidémiologie et Santé des Populations, Institut National de la Santé et de la Recherche Médicale, Université Paris-Saclay, Université de Versailles Saint-Quentin-en-Yvelines, Paris, France; 2 Epidemiology and Public Health Department, Assistance Publique-Hôpitaux de Paris Université Paris-Saclay, Le Kremlin-Bicêtre, France; 3 Ensai, Bruz, France; 4 Institut Pierre Louis d’Epidémiologie et de Santé Publique, Equipe de Recherche en Epidémiologie Sociale, Institut National de la Santé et de la Recherche Médicale, Sorbonne Université, Paris, France

## Abstract

**Importance:**

The long-term consequences of COVID-19 on mental health are a critical issue given the number of people infected with SARS-CoV-2 worldwide since the beginning of the pandemic.

**Objective:**

To investigate the associations between self-reported COVID-19–like symptoms or SARS-CoV-2 seropositivity and subsequent depression or anxiety.

**Design, Setting, and Participants:**

This propensity score–matched cohort study began in May 2020, with follow-ups in November 2020 and July 2021. The study used data from a large, randomly selected, national population–based cohort from France, the EpiCoV (Epidémiologie et Conditions de Vie) study. Of 85 074 individuals 15 years or older who completed the questionnaires at the 3 collection times, 28 568 were excluded because they did not return a blood sample for serologic testing, 1994 because of missing data on outcomes or exposures, and 9252 to respect the temporal sequence (exposure must precede the outcome).

**Exposures:**

Propensity scores based on various socioeconomic, lifestyle, and health variables were computed to match participants who experienced COVID-19–like symptoms between February and November 2020 or showed SARS-CoV-2 seropositivity in November 2020.

**Main Outcomes and Measures:**

Logistic regression models were used to estimate associations between these occurrences and depression or anxiety assessed in July 2021 using the Patient Health Questionnaire 9-item and Generalized Anxiety Disorder 7-item scales, respectively.

**Results:**

Among the 45 260 included participants (mean [SD] age, 51.1 [18.9] years; 52.4% women; 8.0% with depression and 5.3% with anxiety in July 2021), COVID-19–like symptoms were associated with subsequent depression (adjusted odds ratio, 1.70; 95% CI, 1.45-1.99) and anxiety (adjusted OR, 1.57; 95% CI, 1.29-1.92), whereas SARS-CoV-2 seropositivity was not. Furthermore, COVID-19–like symptoms, but not anosmia or dysgeusia alone, were associated with subsequent depression and anxiety in both the seropositive and seronegative subgroups.

**Conclusions and Relevance:**

In this cohort study of more than 45 000 individuals drawn from the French general population, SARS-CoV-2 infection was not found as a risk factor of subsequent depression or anxiety. Moreover, self-reported COVID-19–like symptoms were associated with depression and anxiety assessed at least 8 months later in both seropositive and seronegative subgroups, suggesting that factors other than SARS-CoV-2 infection are implied in this association.

## Key Points

**Question:**

Is SARS-CoV-2 infection associated with subsequent long-term depression or anxiety?

**Findings:**

In this propensity score–matched, population-based cohort study of 45 260 participants, COVID-19–like symptoms, but not SARS-CoV-2 infection, during the first months of the pandemic were associated with an increased occurrence of subsequent depression and anxiety 8 months or more after the occurrence of COVID-19–like symptoms, even when SARS-CoV-2 serologic test results were negative.

**Meaning:**

These findings suggest that SARS-CoV-2 infection is not a risk factor for long-term mental health issues; however, further research is needed to identify factors other than SARS-CoV-2 infection implied in the association between COVID-19–like symptoms and poor mental health outcomes.

## Teaser

This cohort study investigates the association between self-reported COVID-19–like symptoms vs SARS-CoV-2 seropositivity and subsequent depression or anxiety in a nationally representative French population.

## Introduction

Mental health issues are increasingly reported for survivors of SARS-CoV-2 infection.^1,2^ Until recently, the high incidence of depression following SARS-CoV-2 infection has been mostly described for patients hospitalized for COVID-19 without a control condition.^3,4,5^ Three large electronic health record–based cohort studies found higher risks of relapse or a first diagnosis of psychiatric disorders for patients with a diagnosis of COVID-19 than for matched cohorts of patients with other health events.^6,7,8^ The most recent of these cohort studies showed that the increased post–COVID-19 risk of mood and anxiety disorders subsided after 1 to 2 months, with no overall excess during the entire 2-year follow-up.^8^ An increased risk of incident mental health disorders in the year after a polymerase chain reaction (PCR)–confirmed SARS-CoV-2 infection was also found using electronic health records from the US Veterans Health Administration by Xie et al.^9^ These studies reported statistically significant but modest associations between SARS-CoV-2 infection and increased rates of psychiatric disorders.^10^ For example, in the study by Xie et al,^9^ the risk vs contemporary controls was 1.1 per 100 individuals at 1 year (95% CI, 1.0-1.3) for anxiety and 1.5 (95% CI, 1.4-1.7) for depressive disorders.

Various socioeconomic and lifestyle factors can be responsible for confounding (eg, living conditions and occupational status), but little information on such factors is routinely available in electronic health records. Moreover, misclassification may have occurred for both SARS-CoV-2 infection and psychiatric disorders. Indeed, diagnostic tests for SARS-CoV-2 infection were not available at the beginning of the pandemic, and individuals with mild or moderate COVID-19–like symptoms were asked to isolate themselves and self-medicate without seeing a physician. Data from a large number of individuals with SARS-CoV-2 infection may thus be absent from the electronic health records, mostly those with pauci- or asymptomatic disease. Concerning psychiatric disorders, mental health care might have been more accessible to those with known COVID-19 compared with healthy controls without the disease, leading to an overrepresentation of patients with COVID-19 in the electronic health records.^10,11^ Finally, characteristics of the SARS-CoV-2 infection that are not available in electronic health records, for example, COVID-19 with smell and taste loss (suspected to be a sign of SARS-CoV-2 neuroinvasion^12^), could also be involved in the occurrence of psychiatric outcomes, such as the duration or type of symptoms.

Two recent observational studies addressed several of these issues by gathering data from planned cohorts.^13,14^ The initial study had a cross-sectional design and found that the severity of acute COVID-19 (retrospectively self-reported number of days confined to bed because of COVID-19–like symptoms) was associated with anxiety and depression up to 16 months after diagnosis, after adjustment for certain sociodemographic-, lifestyle-, and health-related factors.^13^ Self-reporting of a confirmed positive reverse transcriptase PCR or antibody test result was used as an indicator of a COVID-19 diagnosis. In the other study, similar results were obtained with a longitudinal design using self-reported measures of COVID-19, and SARS-CoV-2 serologic test results were also available but solely for a small subset and, thus, only allowed exploratory analyses, which revealed no association between SARS-CoV-2 infection and mental health outcome.^14^ In the context of these mixed findings from the literature, a recent systematic review highlighted the need for high-quality longitudinal studies with adequate design in this area.^15^

In this study, we aimed to investigate the associations between self-reported COVID-19–like symptoms or SARS-CoV-2 seropositivity and subsequent depression or anxiety using data from a large, randomly selected, national population–based cohort on SARS-CoV-2 pandemic in France, the EpiCoV (Epidémiologie et Conditions de Vie) study. We also investigated the role of anosmia or dysgeusia, as well as the timing and duration of COVID-19–like symptoms, in the occurrence of subsequent depression or anxiety.

## Methods

### Study Data

On May 2, 2020, a letter was sent to a random sample of 371 000 people 15 years or older drawn from the national administrative and tax database (covering 96.4% of the population in France), with an intentional overrepresentation of individuals living in a household below the poverty level. The sampling design is described in detail in Warszawski et al.^16^ Individuals living in a residential care home for the elderly or in prison were excluded. From May 2 to June 2, 2020 (baseline), individuals were invited to answer a questionnaire to collect information on demographic and socioeconomic characteristics, health status (including symptoms potentially linked to COVID-19), and living conditions online or by telephone using a concurrent mixed-mode design (eFigure in Supplement 1).^17^ The 134 391 participants who responded at baseline were contacted to answer new questionnaires in November 2020 (first follow-up) and in July 2021 (second follow-up). At the first follow-up, along with the questionnaire, home capillary blood self-sampling (finger prick) was proposed for SARS-CoV-2 serologic testing using an enzyme-linked immunosorbent assay (ELISA, Euroimmun). The methods for serologic testing are described elsewhere.^17^ Of note, 94.4% of the self-samples were collected before SARS-CoV-2 vaccination became available in France in January 2021. All participants or their legally authorized representatives provided written informed consent to participate in this study. The EpiCov study received approval from the Comité de Protection des Personnes Sud Méditerranée III and the Commission Nationale Informatique et Libertés. The study followed the Strengthening the Reporting of Observational Studies in Epidemiology (STROBE) reporting guideline.^18^

### Main Outcomes

At the second follow-up, participants were asked to respond to the 9 items of the Patient Health Questionnaire (PHQ-9) and the 7 items of the Generalized Anxiety Disorder scale (GAD-7).^19,20^ Scores range from 0 to 27 on the PHQ-9 and from 0 to 21 on the GAD-7, with higher scores indicating higher levels of depression and anxiety. A score of 10 or higher was used for both scales to indicate depression or anxiety.^21,22^

### Exposures

To respect the temporality criteria, only information available at baseline and the first follow-up was used to define the exposures. The presence of COVID-19–like symptoms was defined as the self-reporting of anosmia or dysgeusia or fever with cough, dyspnea, or thoracic oppression at any time from the beginning of the COVID-19 pandemic in France (February 2020) to the first follow-up. The timing and duration of the last occurrence of COVID-19–like symptoms before the first follow-up were also assessed. Serologic status was defined as positive when the ELISA optical density ratio was 0.7 or greater.

### Covariates

Covariate selection to compute propensity scores was based on current literature and knowledge about factors involved in both COVID-19 and depression or anxiety risk. The following sociodemographic covariates were selected: sex, age, immigration status (participants and their parents born in mainland France, participants or their parents born in French oversea territories, participants born in France from parents born outside France, or participants born outside France), highest educational level (none, lower secondary school certificate, professional certificate, higher secondary school certificate, bachelor’s degree or equivalent, or master’s degree or more), main occupational status (employed, student, unemployed, retired, or other, including housemakers), deciles of household income per consumption unit, perceived financial situation (comfortable, decent, short, difficult, or unbearable, without incurring debts), usual residence overcrowded (<1 or ≥1 room per person), household structure (single, couple without children, couple with children, single parent, living with parents, or complex household), living in the usual residence during the first lockdown, access to a private exterior during the first lockdown (balcony or garden), urban density of the area of residence (oversea territories, <2000 urban units, between 2000 and 1 999 999 urban units, or Paris area; an urban unit is a constructed area with <200 m between 2 constructions that comprises at least 2000 habitants), living in a priority neighborhood (area where the poorest inhabitants of the cities live and defined as a high-priority target for city policies), and quartile of the hospitalization rate during the first lockdown in the area of residence. The following health-related covariates were also included: body mass index, perceived health status (very good to good, quite good, or poor to very poor), prepandemic chronic mental or physical conditions, tobacco use (current, past, or never), and alcohol use (daily, often, occasional, rare, or never).

### Statistical Analysis

We used conditional logistic regression to estimate the unadjusted association (crude odds ratio [cOR]) between depression and anxiety at the second follow-up and (1) reporting COVID-19–like symptoms, including anosmia or dysgeusia, before the first follow-up; (2) having a positive SARS-CoV-2 serologic test result at the first follow-up; (3) reporting anosmia or dysgeusia before the first follow-up; (4) reporting COVID-19–like symptoms, including anosmia or dysgeusia, according to the timing of occurrence (in the last 6 months before the first follow-up vs no symptoms or >6 months before the first follow-up vs no symptoms); and (5) reporting COVID-19–like symptoms according to their duration (≤2 weeks vs no symptoms or >2 weeks vs no symptoms). Then, for each of these 5 variables of interest, the R package MatchThem was used to compute propensity scores based on the above described covariates, to perform 1:1 propensity score matching (without replacement) between exposed and nonexposed participants with a caliper distance of 0.2, and to estimate the adjusted associations (adjusted OR [aOR]) with depression and anxiety at the second follow-up.^23,24^ Covariates with the absolute standardized mean difference between the matched exposed and nonexposed participants below 10% were considered to be well balanced (eFigures 2-20 in Supplement 1).^25^ Participants with missing data for variables of interest or outcome measures were excluded from the analyses. Missing data for covariates (up to 4.7%) were imputed using the R package MICE, and all the analyses were performed on each of the 5 imputed data sets and pooled using the pool function of the MatchThem package.^26^

To respect the Bradford Hill criterion^27^ for causation concerning the temporal sequence, we studied the association between the variables of interest described above and the presence of depression or anxiety 8 months later (ie, at the second follow-up) for participants with no history of depression, anxiety, or bipolar disorder at the first follow-up who declared no COVID-19–like symptoms and no positive antigenic or PCR test between the first and second follow-ups. We also performed subgroup analyses according to SARS-CoV-2 serologic test results to examine the association of the presence of COVID-19–like symptoms and, separately, anosmia or dysgeusia when SARS-CoV-2 infection was serologically confirmed and when it was not.

All analyses were performed using R software, version 4.1.0 (R Foundation for Statistical Computing). Study weights (calculated as previously described^17^) were applied to all descriptive analyses to account for the design of EpiCoV and nonparticipating bias. Because it is not technically possible to use study weights in propensity score analyses, we performed sensitivity analyses using weighted logistic regressions adjusted on the same covariates used to compute propensity scores. The statistical significance was set to a 2-sided *P* < .05.

## Results

**Figure 1.**
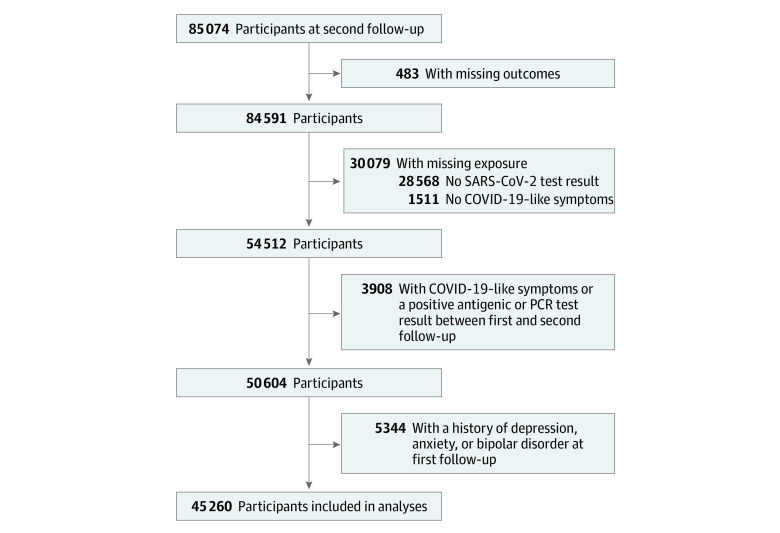
Study Population Flowchart

The participant flowchart is shown in Figure 1, and baseline characteristics of the 45 260 participants (mean [SD] age, 51.1 [18.9] years; 52.4% women and 47.6% men) included in the analyses are shown in eTable 1 in Supplement 1. Eight months later, at the second follow-up, 8.0% (95% CI, 7.6%-8.3%) of participants had a score of 10 or higher on the PHQ-9, and 5.3% (95% CI, 5.0%-5.5%) had a score of 10 or higher on the GAD-7. In total, 11.8% (95% CI, 11.5%-12.2%) of participants reported having had COVID-19–like symptoms before the first follow-up; 5.4% (95% CI, 5.1%-5.6%) reported that their symptoms appeared in the last 6 months before the first follow-up, and 6.5% (95% CI, 6.2%-6.8%) reported symptoms more than 6 months before the first follow-up. Concerning the duration of symptoms, 7.9% (95% CI, 7.6%-8.2%) of participants reported that they lasted 2 weeks or less, and 4.0% (95% CI, 3.7%-4.2%) reported a duration of more than 2 weeks. Anosmia or dysgeusia was reported by 5.2% (95% CI, 4.9%-5.5%) of the participants. Overall, 9.4% (95% CI, 9.1%-9.8%) of the 45 260 participants had positive SARS-CoV-2 serologic test results at the first follow-up. Main outcomes and exposures according to the presence of COVID-19–like symptoms, anosmia or dysgeusia, and SARS-CoV-2 seropositivity are shown in the Table.

**Table.** Main Outcomes and Exposures According to the Presence of COVID-19–Like Symptoms, Anosmia or Dysgeusia, and SARS-CoV-2 Seropositivity in the Study Sample^a^

Outcome	COVID-19–like symptoms		Anosmia or dysgeusia		SARS-CoV-2 seropositivity	
	With (n = 5534)	Without (n = 39 726)	With (n = 2356)	Without (n = 42 904)	Positive (n = 4348)	Negative (n = 40 912)
Main outcomes (second follow-up)						
Depression (PHQ-9 score ≥10)	14.5 (13.2-15.7)	7.1 (6.8-7.5)	12.9 (11.0-14.8)	7.7 (7.3-8.1)	9.1 (7.9-10.3)	7.9 (7.5-8.2)
Anxiety (GAD-7 score ≥10)	8.7 (7.7-9.6)	4.8 (4.5-5.1)	8.7 (7.2-10.2)	5.1 (4.8-5.3)	5.7 (4.8-6.6)	5.2 (4.9-5.5)
Exposures (first follow-up)						
COVID-19–like symptoms^b^	100	0	100	7.0 (6.7-7.3)	40.9 (39.0-42.8)	8.8 (8.5-9.2)
Anosmia or dysgeusia	44.0 (42.3-45.8)	0	100	0	30.4 (28.6-32.2)	2.6 (2.4-2.8)
Date of onset of symptoms before first follow-up, mo						
≤6	45.3 (43.6-47.0)	0	45.3 (42.6-48.0)	3.2 (3.0-3.4)	21.7 (20.1-23.3)	3.7 (3.4-3.9)
>6	54.7 (53.0-56.4)	0	54.7 (52.0-57.4)	3.8 (3.6-4.0)	19.2 (17.7-20.7)	5.2 (4.9-5.4)
Duration of symptoms, wk						
≤2	66.5 (64.9-68.1)	0	57.5 (54.8-60.1)	5.1 (4.9-5.4)	24.3 (22.7-26.0)	6.2 (5.9-6.5)
>2	33.5 (31.9-35.1)	0	42.5 (39.9-45.2)	1.8 (1.7-2.0)	16.6 (15.2-18.0)	2.7 (2.5-2.9)
SARS-CoV-2 seropositivity	32.6 (31.0-34.1)	6.3 (6.0-6.6)	54.9 (52.2-57.6)	6.9 (6.6-7.2)	100	0

Abbreviations: GAD-7, 7-item Generalized Anxiety Disorder scale; PHQ-9, 9-item Patient Health Questionnaire.

^a^ Data are presented as weighted percentages (95% CIs).

^b^ COVID-19–like symptoms are defined as self-reporting of anosmia or dysgeusia or fever with cough, dyspnea, or thoracic oppression.

**Figure 2.**
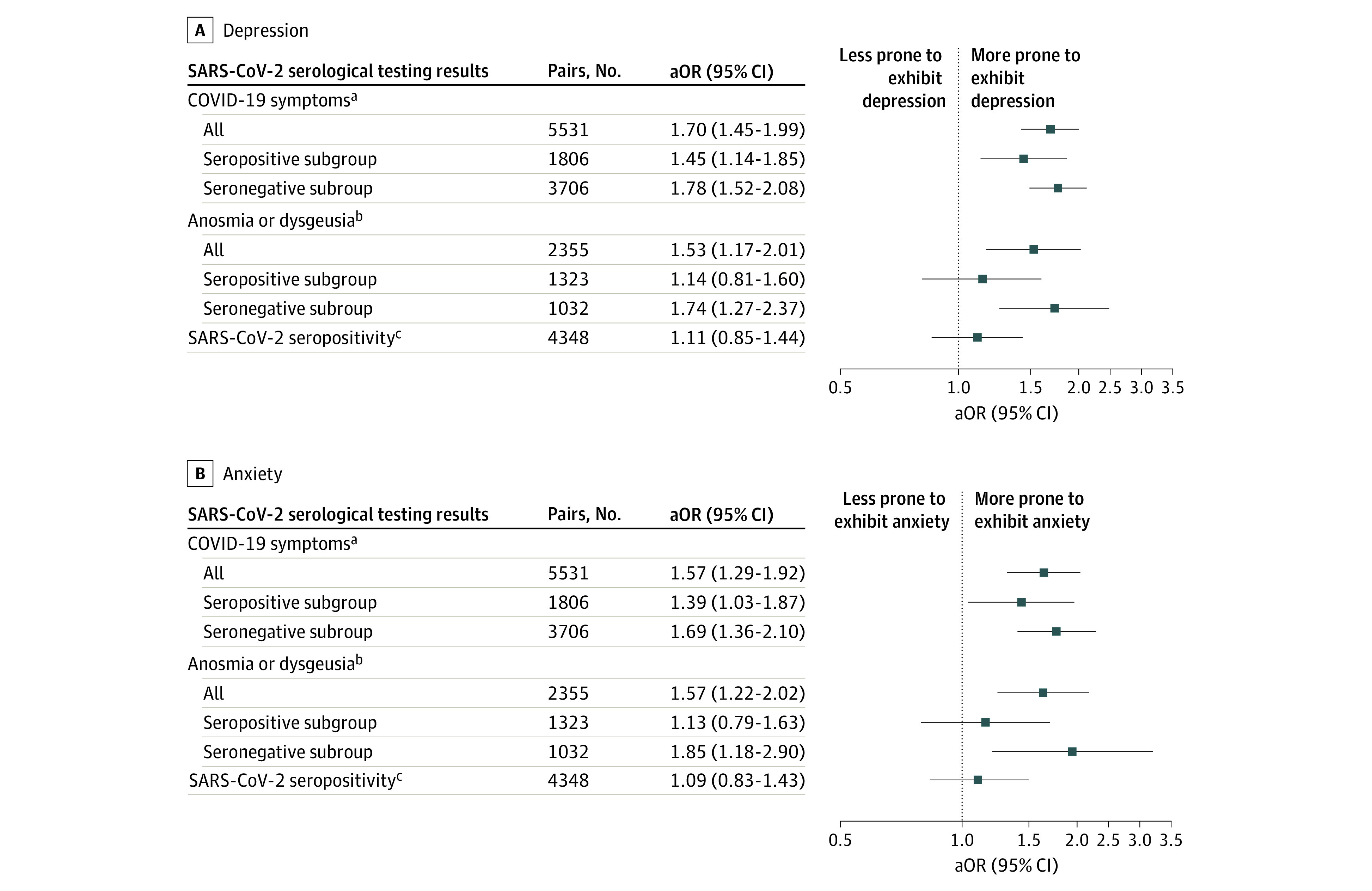
Results of the Propensity Score Analyses Concerning COVID-19–Like Symptoms, SARS-CoV-2 Serologic Test Results, and Anosmia or Dysgeusia The adjusted odds ratios (aORs) were adjusted for the following covariates: sex, age, immigration status, highest educational level, main occupational status, deciles of household income per consumption unit, perceived financial situation, usual residence overcrowded, household structure, living in the usual residence during the first lockdown, access to a private exterior during the first lockdown, urban density of the area of residence, living in a priority neighborhood, quartile of the hospitalization rate during the first lockdown in the area of residence, body mass index, perceived health status, prepandemic chronic mental or physical conditions, tobacco use, and alcohol use. ^a^Reference group for COVID-19 symptoms was no symptoms. ^b^Reference group for anosmia or dysgeusia was no anosmia or dysgeusia. ^c^Reference group for SARS-CoV-2 seropositivity was seronegativity.

**Figure 3.**
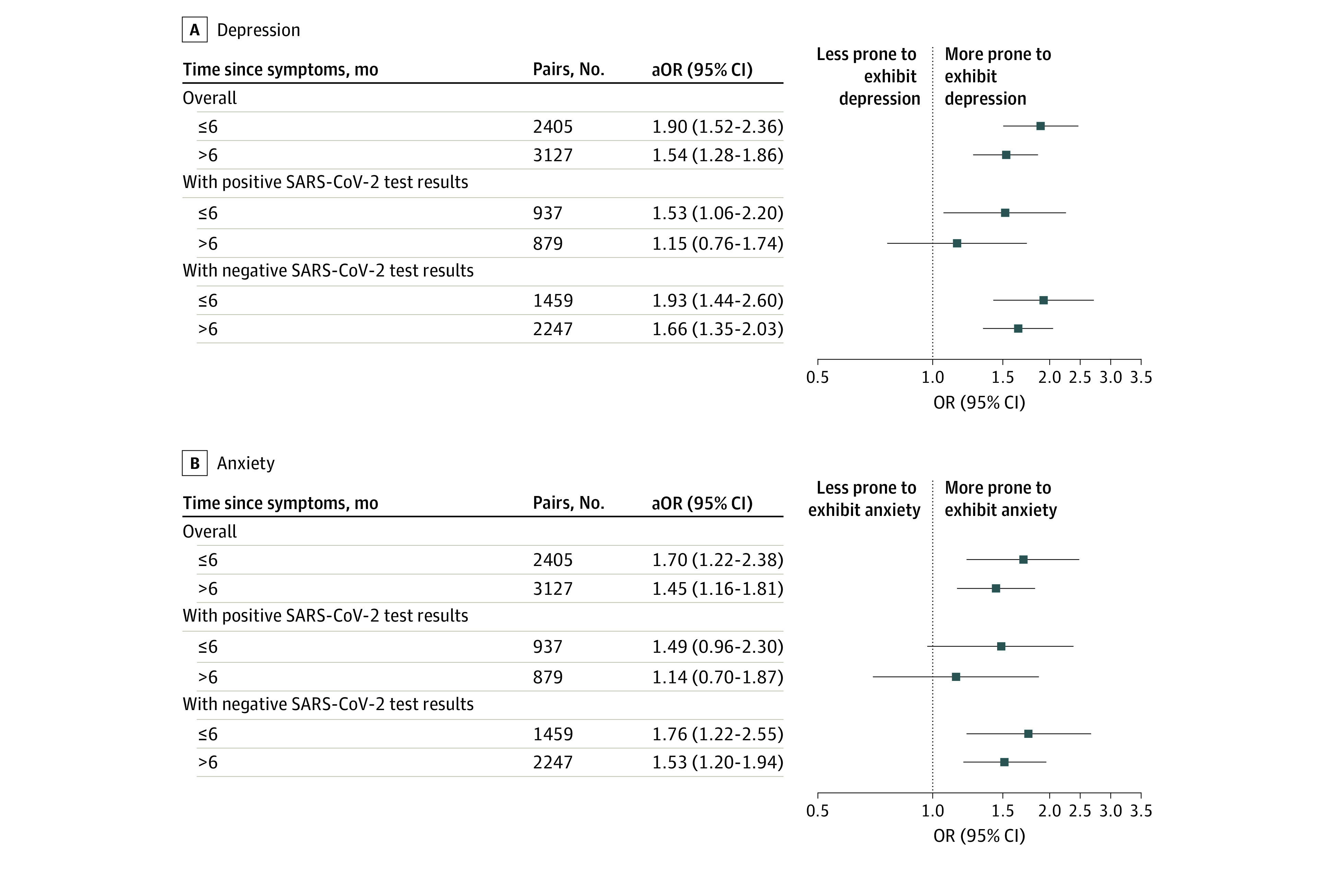
Results of the Propensity Score Analyses Concerning Time of Occurrence of COVID-19–Like Symptoms The adjusted odds ratios (aORs) were adjusted for the following covariates: sex, age, immigration status, highest educational degree, main occupational status, deciles of household income per consumption unit, perceived financial situation, usual residence overcrowded, household structure, living in the usual residence during the first lockdown, access to a private exterior during the first lockdown, urban density of the area of residence, living in a priority neighborhood, quartile of the hospitalization rate during the first lockdown in the area of residence, body mass index, perceived health status, prepandemic chronic mental or physical conditions, tobacco use, and alcohol use. Reference group was no symptoms.

**Figure 4.**
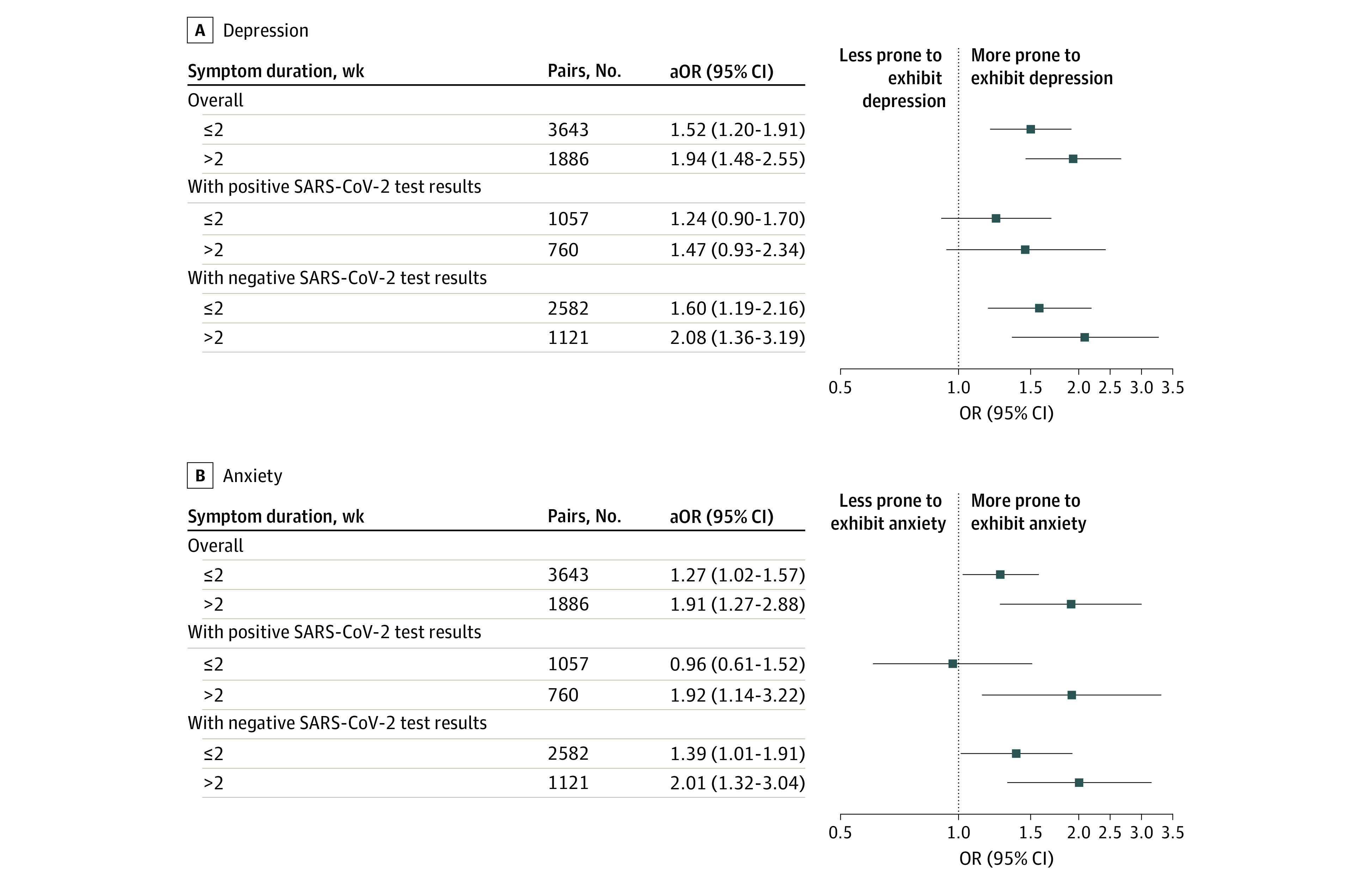
Results of the Propensity Score Analyses Concerning Duration of COVID-19–Like Symptoms The adjusted odds ratios (aORs) were adjusted for the following covariates: sex, age, immigration status, highest educational degree, main occupational status, deciles of household income per consumption unit, perceived financial situation, usual residence overcrowded, household structure, living in the usual residence during the first lockdown, access to a private exterior during the first lockdown, urban density of the area of residence, living in a priority neighborhood, quartile of the hospitalization rate during the first lockdown in the area of residence, body mass index, perceived health status, prepandemic chronic mental or physical conditions, tobacco use, and alcohol use. Reference group was no symptoms.

Results from the propensity score analyses concerning COVID-19–like symptoms, SARS-CoV-2 serologic test results, and anosmia or dysgeusia are presented in Figure 2 and eTable 2 in Supplement 1. Participants reporting COVID-19–like symptoms before the first follow-up were more prone to exhibit depression (cOR, 2.16; 95% CI, 1.97-2.35; aOR, 1.70; 95% CI, 1.45-1.99) or anxiety (cOR, 1.91; 95% CI, 1.71-2.12; aOR, 1.57; 95% CI, 1.29-1.92) at the second follow-up (ie, ≥8 months later). Similar results were obtained when the COVID-19–like symptoms were restricted to anosmia or dysgeusia (cOR, 1.82; 95% CI, 1.60-2.07 and aOR, 1.53; 95% CI, 1.17-2.01 for depression; cOR, 1.87; 95% CI, 1.60-2.16 and aOR, 1.57; 95% CI, 1.22-2.02 for anxiety), but no association was found between positive SARS-CoV-2 serologic test results and depression or anxiety at the second follow-up (cOR, 1.19; 95% CI, 1.06-1.33 and aOR, 1.11; 95% CI, 0.85-1.44 for depression; cOR, 1.12; 95% CI, 0.97-1.28 and aOR, 1.09; 95% CI, 0.83-1.43 for anxiety). In subgroup analyses, COVID-19–like symptoms, but not anosmia or dysgeusia alone, were associated with subsequent depression and anxiety in both the seropositive and seronegative subgroups. The timing of the occurrence and duration of COVID-19–like symptoms showed the same pattern of association with depression and anxiety (Figures 3 and 4; eTables 3 and 4 in Supplement 1). Results from the weighted logistic regressions were similar (eTables 2-4 in Supplement 1).

## Discussion

In this propensity score–matched analysis of data from a cohort of more than 45 000 individuals drawn from the French general population with no history of depression, anxiety, or bipolar disorder, we found that self-reported COVID-19–like symptoms that occurred between February and November 2020 to be associated with depression and anxiety assessed in July 2021 (ie, ≥8 months later). The strength of the measured associations was similar regardless of duration and timing of the symptoms. Restricting the symptoms to self-reported anosmia or dysgeusia, known to be highly specific symptoms of COVID-19, resulted in a similar association with subsequent depression or anxiety. However, when the SARS-CoV-2 infection was objectified by a positive serologic test result in November 2020, no such association was found.

In our opinion, one of the most compelling results of our study was the finding that SARS-CoV-2 seropositivity was not associated with depression or anxiety. To our knowledge, only 1 previous study has reported results from an exploratory analysis using SARS-CoV-2 serologic testing, which are in accordance with ours.^14^ There are several possible explanations for why we observed no association when using serologic testing instead of self-reported symptoms to identify individuals with SARS-CoV-2 infection. First, the ELISA test used in the EpiCoV study had a specificity range of 96.2% to 100% and a sensitivity range of 86.4 to 100%.^28,29,30^ Therefore, a classification bias toward the null is very unlikely. Second, anti–SARS-CoV-2 IgG antibody levels are known to decrease more quickly after infection in individuals with mild or asymptomatic forms.^31^ This finding would suggest that the sensitivity of the serologic test may be lower for less severe and/or less recent infections and that it could have biased the association between serologically defined SARS-CoV-2 infection and mental health problems toward the null. To explore this point, we estimated this association in subgroups according to the duration (as a proxy for severity) and timing of occurrence of the COVID-19–like symptoms. The cORs were similar in the subgroups, suggesting a low likelihood of such bias (eTable 5 in Supplement 1). Third, although serologic test results are an objective indicator of SARS-CoV-2 infection, they do not provide information on the clinical form or its severity. A higher risk of mental health outcomes was observed following severe forms of COVID-19 in several studies.^6,9,13^ In our study, the risk of depression or anxiety was not higher for a duration of symptoms greater than 2 weeks compared with 2 weeks or less relative to the absence of symptoms. However, although COVID-19–like symptoms were associated with mental health outcomes, we found no association with anosmia or dysgeusia for individuals with seropositivity. Therefore, the fact that we found no association with the serologic test results could be explained by differences in the clinical forms of SARS-CoV2 infection among seropositive individuals. To summarize, simply being infected with SARS-Cov-2 did not appear, in our study, to be a risk factor for subsequent mental health disorders, but the presence of COVID-19–like symptoms other than anosmia or dysgeusia was a risk factor among infected individuals.

The results obtained for individuals with SARS-CoV-2 seronegativity similarly show that COVID-19–like symptoms were risk factors for subsequent depression and anxiety in this subgroup. Our results are clearly in accordance with those of the study of Thompson et al,^14^ who noted that self-reporting of COVID-19–like symptoms was associated with poorer mental health when SARS-CoV-2 serologic test results were negative. These investigators did not find this association when SARS-CoV-2 serologic test results were positive, probably because of a lack of power. Another study found that self-reported COVID-19–like symptoms were associated with persistent physical symptoms, whereas laboratory-confirmed COVID-19 was only associated with anosmia.^32^ Both studies^14,32^ suggested mechanisms that may account for these findings. For example, exhibiting COVID-19–like symptoms during the first months of the pandemic may have caused strong concerns about potential health, social, and economic consequences and weakened the psychological balance of certain individuals, leading to poor mental health outcomes, especially if they led to maladaptive health behaviors, such as reduced social contact and/or physical activity.^14,32^

### Limitations

This study has several limitations. One of the limitations was the low response rate to the blood self-sampling for serologic testing; however, data from the national tax database used to randomly select individuals at baseline allowed the use of weighting for nonresponse and thus reduced the potential selection phenomenon. Another limitation was the retrospective assessment of COVID-19–like symptoms, their duration, and the timing of occurrence, which could have resulted in potential recall bias. However, at the time of assessment in November 2020, the beginning of the COVID-19 crisis was less than 9 months earlier, and the exceptional nature of the context may have reinforced the ability of participants to remember events related to this crisis.

## Conclusions

The findings of this longitudinal cohort study on a large sample drawn from the general population in France suggest that SARS-CoV-2 infection is not a risk factor for subsequent depression or anxiety. COVID-19–like symptoms were associated with later depression or anxiety in seronegative individuals, which suggests that factors other than SARS-CoV-2 infection are implied in this association. Further research is needed to identify these factors because they could be targeted to prevent psychological consequences in people who experience these kinds of symptoms and to help patients with depression and anxiety disorders in the current context.

## Notes

Supplement 1. **eTable 1.** Baseline Characteristics of the Study Sample and Subsamples According to the Presence of COVID-19-Like Symptoms, Anosmia/Dysgeusia,or Positive SARS-CoV-2 Serology - Weighted % (95% CI) **eTable 2.** Odds Ratio and 95% Confidence Interval (95% CI) of Depression and Anxiety in July 2021 for Patients With and Without COVID-19 Symptoms Before November 2020, Anosmia and/or Dysgeusia Before November 2020, and Positive SARS-CoV-2 Serology in November 2020, Crude (OR_c_) and Adjusted (OR_a_) for Covariates* Using Propensity-Score Matching (^Ps^) and Conditional Logistic Regression, and Weighted Logistic Regression (^Wlr^) in the Whole Sample and Stratified According to SARS-CoV-2 Serology **eTable 3.** Odds Ratio and 95% Confidence Interval (95% CI) of Depression and Anxiety in July 2021 for Patients With a Time of Occurrence of COVID-19 Symptoms ≤ or >6 Months Before November 2020 Versus Patients Without Symptoms, Crude (OR_c_) and Adjusted (OR_a_) for Covariates Using Propensity-Score Matching and Conditional Logistic Regression (^Ps^), and Weighted Logistic Regression (^Wlr^) in the Entire Sample and Stratified According to SARS-CoV-2 Serology **eTable 4.** Odds Ratio and 95% Confidence Interval of Depression and Anxiety in July 2021 for Patients With a Duration of COVID-19 Symptoms ≤ or >2 Weeks Before November 2020 Versus Patients Without Symptoms, Crude (OR_c_) and Adjusted (OR_a_) for Covariates Using Propensity-Score Matching and Conditional Logistic Regression (^Ps^), and Weighted Logistic Regression (^Wlr^) in the Entire Sample and Stratified According to SARS-CoV-2 Serology **eTable 5.** Crude Odds Ratio (ORc) and 95% Confidence Interval (95% CI) of Depression and Anxiety in July 2021 for Patients With Positive SARS-CoV-2 Serology Versus Patients With Negative Serology Stratified According to the Duration and Time of Occurrence of COVID-19 Symptoms Before November 2020 **eFigure 1.** The EpiCoV Cohort Study Timeline and Data Used in the Present Study **eFigure 2.** Balance in Propensity Score Matching: Absolute Mean Difference (Circle) and Range (Line) Across Imputations for Participants With or Without COVID-19 Symptoms, Before (Unadjusted) and After Matching (Adjusted, N = 5531 Pairs) Performed Within Each 5 Imputed Datasets **eFigure 3.** Balance in Propensity Score Matching: Absolute Mean Difference (Circle) and Range (Line) Across Imputations for Participants With or Without COVID-19 Symptoms, in the Positive SARS-CoV-2 Serology Subsample, Before (Unadjusted) and After Matching (Adjusted, N = 1806 Pairs) Performed Within Each 5 Imputed Datasets **eFigure 4.** Balance in Propensity Score Matching: Absolute Mean Difference (Circle) and Range (Line) Across Imputations for Participants With or Without COVID-19 Symptoms, in the Negative SARS-CoV-2 Serology Subsample, Before (Unadjusted) and After Matching (Adjusted, N = 3706 Pairs) Performed Within Each 5 Imputed Datasets **eFigure 5.** Balance in Propensity Score Matching: Absolute Mean Difference (Circle) and Range (Line) Across Imputations for Participants With or Without Anosmia and/or Dysgeusia, Before (Unadjusted) and After Matching (Adjusted, N = 2355 Pairs) Performed Within Each 5 Imputed Datasets **eFigure 6.** Balance in Propensity Score Matching: Absolute Mean Difference (Circle) and Range (Line) Across Imputations for Participants With or Without Anosmia and/or Dysgeusia, in the Positive SARS-CoV-2 Serology Subsample, Before (Unadjusted) and After Matching (Adjusted, N = 1323 Pairs) Performed Within Each 5 Imputed Datasets **eFigure 7.** Balance in Propensity Score Matching: Absolute Mean Difference (Circle) and Range (Line) Across Imputations for Participants With or Without Anosmia and/or Dysgeusia, in the Negative SARS-CoV-2 Serology Subsample, Before (Unadjusted) and After Matching (Adjusted, N = 1032 Pairs) Performed Within Each 5 Imputed Datasets **eFigure 8.** Balance in Propensity Score Matching: Absolute Mean Difference (Circle) and Range (Line) Across Imputations for Participants With or Without SARS-CoV-2 Serology, Before (Unadjusted) and After Matching (Adjusted, N = 4348 Pairs) Performed Within Each 5 Imputed Datasets **eFigure 9.** Balance in Propensity Score Matching: Absolute Mean Difference (Circle) and Range (Line) Across Imputations for Participants With History of COVID-19 Symptoms ≤6 Months or No Symptom, Before (Unadjusted) and After Matching (Adjusted, N = 2405 Pairs) Performed Within Each 5 Imputed Datasets **eFigure 10.** Balance in Propensity Score Matching: Absolute Mean Difference (Circle) and Range (Line) Across Imputations for Participants With History of COVID-19 Symptoms ≤6 Months or No Symptom, in the Positive SARS-CoV-2 Serology Subsample, Before (Unadjusted) and After Matching (Adjusted, N = 937 Pairs) Performed Within Each 5 Imputed Datasets **eFigure 11.** Balance in Propensity Score Matching: Absolute Mean Difference (Circle) and Range (Line) Across Imputations for Participants With History of COVID-19 Symptoms ≤6 Months or No Symptom, in the Negative SARS-CoV-2 Serology Subsample, Before (Unadjusted) and After Matching (Adjusted, N = 1459 Pairs) Performed Within Each 5 Imputed Datasets **eFigure 12.** Balance in Propensity Score Matching: Absolute Mean Difference (Circle) and Range (Line) Across Imputations for Participants With History of COVID-19 Symptoms >6 Months or No Symptom, Before (Unadjusted) and After Matching (Adjusted, N = 3127 Pairs) Performed Within Each 5 Imputed Datasets **eFigure 13.** Balance in Propensity Score Matching: Absolute Mean Difference (Circle) and Range (Line) Across Imputations for Participants With History of COVID-19 Symptoms >6 Months or No Symptom, in the Positive SARS-CoV-2 Serology Subsample, Before (Unadjusted) and After Matching (Adjusted, N = 879 Pairs) Performed Within Each 5 Imputed Datasets **eFigure 14.** Balance in Propensity Score Matching: Absolute Mean Difference (Circle) and Range (Line) Across Imputations for Participants With History of COVID-19 Symptoms >6 Months or No Symptom, in the Negative SARS-Cov-2 Serology Subsample, Before (Unadjusted) and After Matching (Adjusted, N = 2247 Pairs) Performed Within Each 5 Imputed Datasets **eFigure 15.** Balance in Propensity Score Matching: Absolute Mean Difference (Circle) and Range (Line) Across Imputations for Participants With Duration of COVID-19 Symptoms ≤2 Weeks or No Symptom, Before (Unadjusted) and After Matching (Adjusted, N = 3643 Pairs) Performed Within Each 5 Imputed Datasets **eFigure 16.** Balance in Propensity Score Matching: Absolute Mean Difference (Circle) and Range (Line) Across Imputations for Participants With Duration of COVID-19 Symptoms ≤2 Weeks or No Symptom, in the Positive SARS-CoV-2 Serology Subsample, Before (Unadjusted) and After Matching (Adjusted, N = 1057 Pairs) Performed Within Each 5 Imputed Datasets **eFigure 17.** Balance in Propensity Score Matching: Absolute Mean Difference (Circle) and Range (Line) Across Imputations for Participants With Duration of COVID-19 Symptoms ≤2 Weeks or No Symptom, in the Negative SARS-CoV-2 Serology Subsample, Before (Unadjusted) and After Matching (Adjusted, N = 2582 Pairs) Performed Within Each 5 Imputed Datasets **eFigure 18.** Balance in Propensity Score Matching: Absolute Mean Difference (Circle) and Range (Line) Across Imputations for Participants With Duration of COVID-19 Symptoms >2 Weeks or No Symptom, Before (Unadjusted) and After Matching (Adjusted, N = 1886 Pairs) Performed Within Each 5 Imputed Datasets **eFigure 19.** Balance in Propensity Score Matching: Absolute Mean Difference (Circle) and Range (Line) Across Imputations for Participants With Duration of COVID-19 Symptoms >2 Weeks or No Symptom, in the Positive SARS-CoV-2 Serology Subsample, Before (Unadjusted) and After Matching (Adjusted, N = 760 Pairs) Performed Within Each 5 Imputed Datasets **eFigure 20.** Balance in Propensity Score Matching: Absolute Mean Difference (Circle) and Range (Line) Across Imputations For Participants With Duration of COVID-19 Symptoms >2 Weeks or No Symptom, in the Negative SARS-CoV-2 Serology Subsample, Before (Unadjusted) and After Matching (Adjusted, N = 1121 Pairs) Performed Within Each 5 Imputed Datasets

Supplement 2.
Nonauthor Collaborators

Supplement 3.
Data Sharing Statement
