# Supplementary material for: Comparison of Depression and Anxiety Following Self-reported COVID-19–Like Symptoms vs SARS-CoV-2 Seropositivity in France
Source: JAMA Netw Open. 2023 May 11;6(5):e2312892. doi: 10.1001/jamanetworkopen.2023.12892 (PMC10176124; doi:10.1001/jamanetworkopen.2023.12892)
Supplement: Supplement 1. — eTable 1. Baseline Characteristics of the Study Sample and Subsamples According to the Presence of COVID-19-Like Symptoms, Anosmia/Dysgeusia,or Positive SARS-CoV-2 Serology - Weighted % (95% CI) eTable 2. Odds Ratio and 95% Confidence Interval (95% CI) of Depression and Anxiety in July 2021 for Patients With and Without COVID-19 Symptoms Before November 2020, Anosmia and/or Dysgeusia Before November 2020, and Positive SARS-CoV-2 Serology in November 2020, Crude (ORc) and Adjusted (ORa) for Covariates* Using Propensity-Score Matching (Ps) and Conditional Logistic Regression, and Weighted Logistic Regression (Wlr) in the Whole Sample and Stratified According to SARS-CoV-2 Serology eTable 3. Odds Ratio and 95% Confidence Interval (95% CI) of Depression and Anxiety in July 2021 for Patients With a Time of Occurrence of COVID-19 Symptoms ≤ or >6 Months Before November 2020 Versus Patients Without Symptoms, Crude (ORc) and Adjusted (ORa) for Covariates Using Propensity-Score Matching and Conditional Logistic Regression (Ps), and Weighted Logistic Regression (Wlr) in the Entire Sample and Stratified According to SARS-CoV-2 Serology eTable 4. Odds Ratio and 95% Confidence Interval of Depression and Anxiety in July 2021 for Patients With a Duration of COVID-19 Symptoms ≤ or >2 Weeks Before November 2020 Versus Patients Without Symptoms, Crude (ORc) and Adjusted (ORa) for Covariates Using Propensity-Score Matching and Conditional Logistic Regression (Ps), and Weighted Logistic Regression (Wlr) in the Entire Sample and Stratified According to SARS-CoV-2 Serology eTable 5. Crude Odds Ratio (ORc) and 95% Confidence Interval (95% CI) of Depression and Anxiety in July 2021 for Patients With Positive SARS-CoV-2 Serology Versus Patients With Negative Serology Stratified According to the Duration and Time of Occurrence of COVID-19 Symptoms Before November 2020 eFigure 1. The EpiCoV Cohort Study Timeline and Data Used in the Present Study eFigure 2. Balance in Propensity Score Matchin [file jamanetwopen-e2312892-s001.pdf]

Supplementary Online Content

Rouquette A, Descarpentry A, Dione F, et al; EpiCoV Study Group. Comparison of depression and anxiety following self-reported COVID-19–like symptoms vs SARS-CoV-2 seropositivity in France. *JAMA Netw Open*. 2023;6(5):e2312892. doi:10.1001/jamanetworkopen.2023.12892

eTable 1. Baseline Characteristics of the Study Sample and Subsamples According to the Presence of COVID-19-Like Symptoms, Anosmia/Dysgeusia,or Positive SARS-CoV-2 Serology - Weighted % (95%CI)

eTable 2. Odds Ratio and 95% Confidence Interval (95% CI) of Depression and Anxiety in July 2021 for Patients With and Without COVID-19 Symptoms Before November 2020, Anosmia and/or Dysgeusia Before November 2020, and Positive SARS-CoV-2 Serology in November 2020, Crude (OR_c) and Adjusted (OR_a) for Covariates* Using Propensity-Score Matching (^{Ps}) and Conditional Logistic Regression, and Weighted Logistic Regression (^{Wlr}) in the Whole Sample and Stratified According to SARS-CoV-2 Serology

eTable 3. Odds Ratio and 95% Confidence Interval (95% CI) of Depression and Anxiety in July 2021 for Patients With a Time of Occurrence of COVID-19 Symptoms ≤ or > 6 Months Before November 2020 Versus Patients Without Symptoms, Crude (OR_c) and Adjusted (OR_a) for Covariates Using Propensity-Score Matching and Conditional Logistic Regression (^{Ps}), and Weighted Logistic Regression (^{Wlr}) in the Entire Sample and Stratified According to SARS-CoV-2 Serology

eTable 4. Odds Ratio and 95% Confidence Interval of Depression and Anxiety in July 2021 for Patients With a Duration of COVID-19 Symptoms ≤ or > 2 Weeks Before November 2020 Versus Patients Without Symptoms, Crude (OR_c) and Adjusted (OR_a) for Covariates Using Propensity-Score Matching and Conditional Logistic Regression (^{Ps}), and Weighted Logistic Regression (^{Wlr}) in the Entire Sample and Stratified According to SARS-CoV-2 Serology

eTable 5. Crude Odds Ratio (OR_c) and 95% Confidence Interval (95%CI) of Depression and Anxiety in July 2021 for Patients With Positive SARS-CoV-2 Serology Versus Patients With Negative Serology Stratified According to the Duration and Time of Occurrence of COVID-19 Symptoms Before November 2020

eFigure 1. The EpiCoV Cohort Study Timeline and Data Used in the Present Study

eFigure 2. Balance in Propensity Score Matching: Absolute Mean Difference (Circle) and Range (Line) Across Imputations for Participants With or Without COVID-19 Symptoms, Before (Unadjusted) and After Matching (Adjusted, N=5531 Pairs) Performed Within Each 5 Imputed Datasets

eFigure 3. Balance in Propensity Score Matching: Absolute Mean Difference (Circle) and Range (Line) Across Imputations for Participants With or Without COVID-19 Symptoms, in the Positive SARS-CoV-2 Serology Subsample, Before (Unadjusted) and After Matching (Adjusted, N=1806 Pairs) Performed Within Each 5 Imputed Datasets

eFigure 4. Balance in Propensity Score Matching: Absolute Mean Difference (Circle) and Range (Line) Across Imputations for Participants With or Without COVID-19 Symptoms, in the Negative SARS-CoV-2 Serology Subsample, Before (Unadjusted) and After Matching (Adjusted, N=3706 Pairs) Performed Within Each 5 Imputed Datasets

© 2023 Rouquette A et al. *JAMA Network Open.*

eFigure 5. Balance in Propensity Score Matching: Absolute Mean Difference (Circle) and Range (Line) Across Imputations for Participants With or Without Anosmia and/or Dysgeusia, Before (Unadjusted) and After Matching (Adjusted, N=2355 Pairs) Performed Within Each 5 Imputed Datasets
eFigure 6. Balance in Propensity Score Matching: Absolute Mean Difference (Circle) and Range (Line) Across Imputations for Participants With or Without Anosmia and/or Dysgeusia, in the Positive SARS-CoV-2 Serology Subsample, Before (Unadjusted) and After Matching (Adjusted, N=1323 Pairs) Performed Within Each 5 Imputed Datasets
eFigure 7. Balance in Propensity Score Matching: Absolute Mean Difference (Circle) and Range (Line) Across Imputations for Participants With or Without Anosmia and/or Dysgeusia, in the Negative SARS-CoV-2 Serology Subsample, Before (Unadjusted) and After Matching (Adjusted, N=1032 Pairs) Performed Within Each 5 Imputed Datasets
eFigure 8. Balance in Propensity Score Matching: Absolute Mean Difference (Circle) and Range (Line) Across Imputations for Participants With or Without SARS-CoV-2 Serology, Before (Unadjusted) and After Matching (Adjusted, N=4348 Pairs) Performed Within Each 5 Imputed Datasets
eFigure 9. Balance in Propensity Score Matching: Absolute Mean Difference (Circle) and Range (Line) Across Imputations for Participants With History of COVID-19 Symptoms ≤ 6 Months or No Symptom, Before (Unadjusted) and After Matching (Adjusted, N=2405 Pairs) Performed Within Each 5 Imputed Datasets
eFigure 10. Balance in Propensity Score Matching: Absolute Mean Difference (Circle) and Range (Line) Across Imputations for Participants With History of COVID-19 Symptoms ≤ 6 Months Or No Symptom, in the Positive SARS-CoV-2 Serology Subsample, Before (Unadjusted) and After Matching (Adjusted, N=937 Pairs) Performed Within Each 5 Imputed Datasets
eFigure 11. Balance in Propensity Score Matching: Absolute Mean Difference (Circle) and Range (Line) Across Imputations for Participants With History of COVID-19 Symptoms ≤ 6 Months or No Symptom, in the Negative SARS-CoV-2 Serology Subsample, Before (Unadjusted) and After Matching (Adjusted, N=1459 Pairs) Performed Within Each 5 Imputed Datasets
eFigure 12. Balance in Propensity Score Matching: Absolute Mean Difference (Circle) and Range (Line) Across Imputations for Participants With History of COVID-19 Symptoms > 6 Months or No Symptom, Before (Unadjusted) and After Matching (Adjusted, N=3127 Pairs) Performed Within Each 5 Imputed Datasets
eFigure 13. Balance in Propensity Score Matching: Absolute Mean Difference (Circle) and Range (Line) Across Imputations for Participants With History of COVID-19 Symptoms > 6 Months or No Symptom, in the Positive SARS-CoV-2 Serology Subsample, Before (Unadjusted) and After Matching (Adjusted, N=879 Pairs) Performed Within Each 5 Imputed Datasets
eFigure 14. Balance in Propensity Score Matching: Absolute Mean Difference (Circle) and Range (Line) Across Imputations for Participants With History of COVID-19 Symptoms > 6 Months or No Symptom, in the Negative SARS-Cov-2 Serology Subsample, Before (Unadjusted) and After Matching (Adjusted, N=2247 Pairs) Performed Within Each 5 Imputed Datasets
eFigure 15. Balance in Propensity Score Matching: Absolute Mean Difference (Circle) and Range (Line) Across Imputations for Participants With Duration of COVID-19 Symptoms ≤ 2 Weeks or No Symptom, Before (Unadjusted) and After Matching (Adjusted, N=3643 Pairs) Performed Within Each 5 Imputed Datasets

© 2023 Rouquette A et al. *JAMA Network Open.*

eFigure 16. Balance in Propensity Score Matching: Absolute Mean Difference (Circle) and Range (Line) Across Imputations for Participants With Duration of COVID-19 Symptoms ≤ 2 Weeks or No Symptom, in the Positive SARS-CoV-2 Serology Subsample, Before (Unadjusted) and After Matching (Adjusted, N=1057 Pairs) Performed Within Each 5 Imputed Datasets
eFigure 17. Balance in Propensity Score Matching: Absolute Mean Difference (Circle) and Range (Line) Across Imputations for Participants With Duration of COVID-19 Symptoms ≤ 2 Weeks or No Symptom, in the Negative SARS-CoV-2 Serology Subsample, Before (Unadjusted) and After Matching (Adjusted, N=2582 Pairs) Performed Within Each 5 Imputed Datasets
eFigure 18. Balance in Propensity Score Matching: Absolute Mean Difference (Circle) and Range (Line) Across Imputations for Participants With Duration of COVID-19 Symptoms > 2 Weeks or No Symptom, Before (Unadjusted) and After Matching (Adjusted, N=1886 Pairs) Performed Within Each 5 Imputed Datasets
eFigure 19. Balance in Propensity Score Matching: Absolute Mean Difference (Circle) and Range (Line) Across Imputations for Participants With Duration of COVID-19 Symptoms > 2 Weeks or No Symptom, in the Positive SARS-CoV-2 Serology Subsample, Before (Unadjusted) and After Matching (Adjusted, N=760 Pairs) Performed Within Each 5 Imputed Datasets
eFigure 20. Balance in Propensity Score Matching: Absolute Mean Difference (Circle) and Range (Line) Across Imputations For Participants With Duration of COVID-19 Symptoms > 2 Weeks or No Symptom, in the Negative SARS-CoV-2 Serology Subsample, Before (Unadjusted) and After Matching (Adjusted, N=1121 Pairs) Performed Within Each 5 Imputed Datasets

This supplementary material has been provided by the authors to give readers additional information about their work.

© 2023 Rouquette A et al. *JAMA Network Open.*

eTable 1. Baseline Characteristics of the Study Sample and Subsamples According to the Presence of COVID-19-Like Symptoms, Anosmia/Dysgeusia,or Positive SARS-CoV-2 Serology - Weighted % (95%CI)

	All (N = 45 260)	With COVID-19 symptoms (N = 5534)	With anosmia/dysgeusia (N = 2356)	With positive SARS-CoV-2 serology (N = 4348)
Woman	52.4 (51.7; 53.0)	56.5 (54.8; 58.2)	58.2 (55.6; 60.9)	54.7 (52.8; 56.7)
Age (years)				
15 – 25	11.6 (11.2; 12.0)	17.0 (15.7; 18.3)	16.0 (14.0; 18.0)	15.6 (14.2; 17.1)
26 – 45	27.4 (26.8; 27.9)	37.2 (35.6; 38.9)	35.8 (33.2; 38.3)	33.3 (31.4; 35.1)
46 – 65	34.5 (34.0; 35.1)	33.2 (31.6; 34.7)	35.0 (32.5; 37.6)	32.1 (30.4; 33.9)
> 65	26.5 (25.9; 27.1)	12.6 (11.3; 13.9)	13.2 (11.1; 15.2)	18.9 (17.2; 20.7)
Immigration status				
Participant and parents born in mainland France	82.1 (81.6; 82.7)	78.4 (76.8; 80.0)	73.7 (71.0; 76.4)	74.9 (73.0; 76.8)
Participant and/or parents born in overseas territories	2.0 (1.8; 2.2)	1.8 (1.4; 2.3)	2.4 (1.6; 3.2)	2.8 (2.2; 3.4)
Native from France, parents born outside France	8.5 (8.1; 8.9)	10.5 (9.4; 11.7)	11.4 (9.6; 13.2)	12.3 (10.9; 13.7)
Born outside France	7.4 (7.0; 7.8)	9.3 (8.0; 10.5)	12.6 (10.2; 14.9)	10.0 (8.5; 11.4)
Highest educational diploma				
None	7.8 (7.3; 8.3)	6.3 (5.1; 7.6)	9.0 (6.7; 11.4)	8.0 (6.4; 9.6)
Lower secondary school certificate	13.3 (12.8; 13.8)	10.5 (9.3; 11.8)	10.8 (8.9; 12.8)	11.0 (9.5; 12.4)
Professional certificate	19.2 (18.7; 19.7)	14.2 (13.0; 15.4)	14.4 (12.6; 16.3)	14.3 (13.0; 15.6)
Higher secondary school certificate	19.5 (19.1; 20.0)	21.1 (19.7; 22.4)	19.3 (17.3; 21.3)	21.1 (19.5; 22.7)
Bachelor’s degree or equivalent	25.5 (25.0; 25.9)	29.4 (27.9; 30.8)	28.2 (26.0; 30.5)	28.4 (26.7; 30.0)
Master’s degree or higher	14.7 (14.4; 15.1)	18.4 (17.3; 19.6)	18.2 (16.5; 19.9)	17.3 (16.0; 18.6)
Main occupational status before the first lockdown				
Employed	48.3 (47.6; 48.9)	57.6 (55.9; 59.3)	57.3 (54.6; 60.0)	53.6 (51.6; 55.6)
Student	9.0 (8.7; 9.3)	13.9 (12.7; 15.0)	12.5 (10.8; 14.2)	12.8 (11.5; 14.0)
Unemployed	4.4 (4.1; 4.6)	5.1 (4.2; 5.9)	4.5 (3.3; 5.8)	4.2 (3.3; 5.2)
Retired	31.0 (30.4; 31.6)	15.8 (14.5; 17.1)	16.8 (14.7; 19.0)	21.5 (19.7; 23.3)
Other situation	7.4 (7.1; 7.8)	7.7 (6.6; 8.7)	8.8 (7.0; 10.6)	7.9 (6.8; 9.0)
Household income per consumption unit				
First and second decile (poorest households)	13.4 (12.9; 14.0)	15.0 (13.5; 16.4)	15.9 (13.5; 18.3)	14.8 (13.1; 16.5)
Third and fourth deciles	16.3 (15.7; 16.8)	16.8 (15.3; 18.3)	15.5 (13.2; 17.9)	14.8 (13.2; 16.5)
Fifth and sixth deciles (median households)	20.1 (19.5; 20.6)	19.2 (17.7; 20.6)	18.8 (16.6; 21.0)	19.2 (17.5; 20.9)

© 2023 Rouquette A et al. *JAMA Network Open.*

Seventh and eighth deciles	24.0 (23.5; 24.5)	24.2 (22.8; 25.6)	24.3 (22.0; 26.5)	24.9 (23.2; 26.5)
Ninth and tenth deciles (wealthiest households)	26.2 (25.7; 26.7)	24.8 (23.5; 26.1)	25.5 (23.5; 27.5)	26.3 (24.8; 27.8)
Perceived financial situation				
Comfortable	16.7 (16.3; 17.1)	16.8 (15.6; 17.9)	16.7 (14.9; 18.4)	17.8 (16.4; 19.1)
Decent	44.1 (43.5; 44.7)	40.6 (38.9; 42.2)	41.1 (38.4; 43.7)	43.6 (41.7; 45.6)
Tight	30.7 (30.1; 31.3)	31.6 (30.0; 33.3)	32.2 (29.6; 34.8)	28.7 (26.8; 30.6)
Difficult or unbearable without incurring debt	8.5 (8.1; 8.9)	11.0 (9.7; 12.3)	10.1 (8.1; 12.1)	9.9 (8.4; 11.5)
Usual residence overcrowded	6.1 (5.8; 6.5)	10.1 (8.9; 11.3)	11.6 (9.7; 13.6)	10.2 (8.8; 11.6)
Household structure				
Single	16.4 (15.9; 17.0)	14.9 (13.5; 16.3)	13.8 (11.6; 16.0)	12.3 (10.8; 13.7)
Couple without children	32.7 (32.2; 33.3)	23.5 (22.1; 24.9)	23.4 (21.2; 25.6)	25.6 (23.9; 27.3)
Couple with children	28.6 (28.1; 29.1)	33.9 (32.4; 35.5)	35.2 (32.7; 37.7)	33.8 (32.0; 35.7)
Single-parent family	7.0 (6.7; 7.3)	8.0 (7.1; 8.9)	8.0 (6.4; 9.5)	7.9 (6.8; 9.0)
Child living at family home	8.1 (7.8; 8.5)	11.4 (10.3; 12.6)	10.6 (8.9; 12.2)	11.1 (9.9; 12.3)
Complex household	7.1 (6.8; 7.5)	8.2 (7.3; 9.2)	9.1 (7.6; 10.6)	9.2 (8.0; 10.4)
Usual residence during the first lockdown	95.1 (94.8; 95.3)	93.4 (92.6; 94.2)	93.8 (92.6; 94.9)	93.3 (92.3; 94.2)
Residence with private exterior during the first lockdown	90.7 (90.3; 91.1)	88.8 (87.5; 90.0)	88.0 (86.0; 90.1)	89.4 (88.1; 90.8)
Urban density of area of residence (urban units)				
Oversea territories	1.3 (1.2; 1.4)	1.0 (0.7; 1.2)	0.9 (0.5; 1.3)	1.5 (1.1; 1.9)
Rural	24.3 (23.8; 24.8)	20.9 (19.5; 22.2)	19.6 (17.6; 21.7)	19.8 (18.3; 21.2)
Between 2000 and 1 999 999	59.7 (59.1; 60.3)	57.4 (55.7; 59.0)	53.7 (51.0; 56.4)	55.2 (53.3; 57.2)
Paris area	14.7 (14.3; 15.2)	20.8 (19.4; 22.2)	25.8 (23.3; 28.2)	23.5 (21.8; 25.2)
Priority neighbourhood	3.9 (3.6; 4.2)	4.4 (3.4; 5.3)	5.8 (4.0; 7.5)	5.7 (4.6; 6.9)
Body mass index (kg/m²)				
< 18.5	3.5 (3.2; 3.7)	4.5 (3.7; 5.2)	4.3 (3.1; 5.6)	3.7 (2.9; 4.4)
≥ 18.5 and < 25	51.8 (51.1; 52.4)	54.3 (52.5; 56.0)	55.1 (52.4; 57.9)	52.6 (50.6; 54.6)
≥ 25 and < 30	30.8 (30.3; 31.4)	28.8 (27.2; 30.4)	27.9 (25.4; 30.4)	29.4 (27.6; 31.3)
≥ 30	13.9 (13.5; 14.4)	12.5 (11.3; 13.7)	12.7 (10.7; 14.6)	14.3 (12.7; 15.8)
Perceived health status				
Well to very well	80.3 (79.8; 80.9)	79.5 (78.0; 81.1)	81.6 (79.2; 84.0)	83.4 (81.8; 85.1)
Quite well	17.0 (16.5; 17.5)	17.4 (16.0; 18.8)	15.4 (13.2; 17.6)	14.4 (12.8; 15.9)
Poor to very poor	2.7 (2.4; 2.9)	3.1 (2.4; 3.8)	3.0 (1.8; 4.3)	2.2 (1.4; 3.0)
Pre-pandemic chronic somatic condition	29.7 (29.1; 30.3)	25.8 (24.2; 27.3)	24.4 (22.1; 26.8)	26.3 (24.4; 28.1)

© 2023 Rouquette A et al. *JAMA Network Open.*

Pre-pandemic chronic psychiatric condition (other than mood disorders)	1.2 (1.0; 1.3)	1.2 (0.9; 1.5)	1.0 (0.6; 1.4)	1.0 (0.6; 1.4)
Tobacco use				
Never	49.5 (48.9; 50.2)	48.2 (46.5; 49.9)	49.6 (46.9; 52.3)	58.8 (56.8; 60.7)
Former	32.1 (31.6; 32.7)	31.4 (29.8; 33.0)	32.7 (30.2; 35.3)	29.9 (28.2; 31.7)
Current	18.3 (17.8; 18.8)	20.4 (18.9; 21.8)	17.7 (15.7; 19.7)	11.3 (10.1; 12.5)
Alcohol use				
Never	28.2 (27.6; 28.8)	29.7 (28.1; 31.4)	32.2 (29.5; 35.0)	31.3 (29.3; 33.3)
Rare	13.7 (13.3; 14.1)	13.5 (12.3; 14.7)	13.4 (11.6; 15.1)	15.0 (13.6; 16.5)
Occasional	23.9 (23.4; 24.4)	24.1 (22.7; 25.5)	22.6 (20.4; 24.7)	22.4 (20.9; 24.0)
Often	23.5 (23.0; 24.0)	24.4 (23.0; 25.7)	23.8 (21.7; 25.9)	22.6 (21.1; 24.1)
Daily	10.7 (10.4; 11.1)	8.3 (7.4; 9.2)	8.0 (6.7; 9.3)	8.6 (7.5; 9.7)

© 2023 Rouquette A et al. *JAMA Network Open.*

eTable 2. Odds Ratio and 95% Confidence Interval (95% CI) of Depression and Anxiety in July 2021 for Patients With and Without COVID-19 Symptoms Before November 2020, Anosmia and/or Dysgeusia Before November 2020, and Positive SARS-CoV-2 Serology in November 2020, Crude (OR_c) and Adjusted (OR_a) for Covariates* Using Propensity-Score Matching (^{Ps}) and Conditional Logistic Regression, and Weighted Logistic Regression (^{Wlr}) in the Whole Sample and Stratified According to SARS-CoV-2 Serology

		N before matching	N Number of pairs after matching	Depression			Anxiety		
				OR_c [95% CI]	OR_a [95% CI] ^{ps}	OR_a [95% CI] ^{wlr}	OR_c [95% CI]	OR_a [95% CI] ^{ps}	OR_a [95% CI] ^{wlr}
All		**45 260**							
COVID-19 symptoms	No	39 726	5531	1 (Reference)	1 (Reference)	1 (Reference)	1 (Reference)	1 (Reference)	1 (Reference)
	Yes	5534		2.16 [1.97-2.35]	1.70 [1.45-1.99]	1.79 [1.58-2.02]	1.91 [1.71-2.12]	1.57 [1.29-1.92]	1.51 [1.31-1.74]
Anosmia/dysgeusia	No	42 904	2355	1 (Reference)	1 (Reference)	1 (Reference)	1 (Reference)	1 (Reference)	1 (Reference)
	Yes	2356		1.82 [1.60-2.07]	1.53 [1.17-2.01]	1.51 [1.26-1.82]	1.87 [1.60-2.16]	1.57 [1.22-2.02]	1.51 [1.23-1.84]
SARS-CoV-2 serology	No	40 912	4348	1 (Reference)	1 (Reference)	1 (Reference)	1 (Reference)	1 (Reference)	1 (Reference)
	Yes	4348		1.19 [1.06-1.33]	1.11 [0.85-1.44]	1.08 [0.92-1.26]	1.12 [0.97-1.28]	1.09 [0.83-1.43]	1.00 [0.84-1.19]
Positive SARS-CoV-2 serology		**4348**							
COVID-19 symptoms	No	2521	1806	1 (Reference)	1 (Reference)	1 (Reference)	1 (Reference)	1 (Reference)	1 (Reference)
	Yes	1827		1.66 [1.34-2.06]	1.45 [1.14-1.85]	1.35 [1.01-1.82]	1.54 [1.18-2.00]	1.39 [1.03-1.87]	1.41 [1.01-1.96]
Anosmia/dysgeusia	No	3025	1323	1 (Reference)	1 (Reference)	1 (Reference)	1 (Reference)	1 (Reference)	1 (Reference)
	Yes	1323		1.39 [1.11-1.74]	1.14 [0.81-1.60]	1.10 [0.82-1.48]	1.34 [1.02-1.75]	1.13 [0.79-1.63]	0.99 [0.72-1.38]
Negative SARS-CoV-2 serology		**40 912**							
COVID-19 symptoms	No	37 205	3706	1 (Reference)	1 (Reference)	1 (Reference)	1 (Reference)	1 (Reference)	1 (Reference)
	Yes	3707		2.38 [2.15-2.63]	1.78 [1.52-2.08]	1.92 [1.67-2.22]	2.10 [1.85-2.37]	1.69 [1.36-2.10]	1.58 [1.34-1.86]
Anosmia/dysgeusia	No	39 879	1032	1 (Reference)	1 (Reference)	1 (Reference)	1 (Reference)	1 (Reference)	1 (Reference)
	Yes	1033		2.24 [1.87-2.67]	1.74 [1.27-2.37]	1.80 [1.38-2.36]	2.50 [2.04-3.04]	1.85 [1.18-2.90]	2.05 [1.57-2.67]

*Covariates used to compute propensity score and as adjustment covariates in weighted logistic regressions were: gender, age, immigration status, highest educational degree, main occupational status, deciles of household income per consumption unit, perceived financial situation, usual residence overcrowded, household structure, living in the usual residence during the first lockdown, access to a private exterior during the first lockdown, urban density of the area of residence, living in a "priority neighbourhood", quartile of the hospitalisation rate during the first lockdown in the area of residence, body mass index, perceived health status, pre-pandemic chronic mental or physical conditions, tobacco use, and alcohol use

© 2023 Rouquette A et al. *JAMA Network Open.*

eTable 3. Odds Ratio and 95% Confidence Interval (95% CI) of Depression and Anxiety in July 2021 for Patients With a Time of Occurrence of COVID-19 Symptoms ≤ or > 6 Months Before November 2020 Versus Patients Without Symptoms, Crude (OR_c) and Adjusted (OR_a) for Covariates Using Propensity-Score Matching and Conditional Logistic Regression (^{Ps}), and Weighted Logistic Regression (^{Wlr}) in the Entire Sample and Stratified According to SARS-CoV-2 Serology

		N before matching	Number of pairs after matching	Depression			Anxiety		
				OR_c [95% CI]	OR_a [95% CI] ^{ps}	OR_a [95% CI] ^{wlr}	OR_c [95% CI]	OR_a [95% CI] ^{ps}	OR_a [95% CI] ^{wlr}
All		**45 260**							
≤ 6 months	No symptom	39 726	2405	1 (Reference)	1 (Reference)	1 (Reference)	1 (Reference)	1 (Reference)	1 (Reference)
	Yes	2406		2.53 [2.25-2.85]	1.90 [1.52-2.36]	2.14 [1.81-2.54]	2.21 [1.91-2.55]	1.70 [1.22-2.38]	1.74 [1.44-2.10]
> 6 months	No symptom	39 726	3127	1 (Reference)	1 (Reference)	1 (Reference)	1 (Reference)	1 (Reference)	1 (Reference)
	Yes	3128		1.88 [1.67-2.11]	1.54 [1.28-1.86]	1.50 [1.28-1.75]	1.68 [1.46-1.94]	1.45 [1.16-1.81]	1.31 [1.08-1.58]
Positive SARS-CoV-2 serology		**4348**							
≤ 6 months	No symptom	2521	937	1 (Reference)	1 (Reference)	1 (Reference)	1 (Reference)	1 (Reference)	1 (Reference)
	Yes	947		1.89 [1.47-2.43]	1.53 [1.06-2.20]	1.82 [1.25-2.65]	1.79 [1.32-2.42]	1.49 [0.96-2.30]	1.63 [1.11-2.39]
> 6 months	No symptom	2521	879	1 (Reference)	1 (Reference)	1 (Reference)	1 (Reference)	1 (Reference)	1 (Reference)
	Yes	880		1.42 [1.07-1.87]	1.15 [0.76-1.74]	1.01 [0.70-1.46]	1.27 [0.90-1.78]	1.14 [0.70-1.87]	1.15 [0.76-1.74]
Negative SARS-CoV-2 serology		**40 912**							
≤ 6 months	No symptom	37 205	1459	1 (Reference)	1 (Reference)	1 (Reference)	1 (Reference)	1 (Reference)	1 (Reference)
	Yes	1459		2.93 [2.53-3.37]	1.93 [1.44-2.60]	2.36 [1.93-2.89]	2.49 [2.09-2.95]	1.76 [1.22-2.55]	1.88 [1.49-2.36]
> 6 months	No symptom	37 205	2247	1 (Reference)	1 (Reference)	1 (Reference)	1 (Reference)	1 (Reference)	1 (Reference)
	Yes	2248		2.05 [1.79-2.33]	1.66 [1.35-2.03]	1.61 [1.34-1.93]	1.85 [1.57-2.16]	1.53 [1.20-1.94]	1.36 [1.01-1.68]

*Covariates used to compute propensity score and as adjustment covariates in weighted logistic regressions were: gender, age, immigration status, highest educational degree, main occupational status, deciles of household income per consumption unit, perceived financial situation, usual residence overcrowded, household structure, living in the usual residence during the first lockdown, access to a private exterior during the first lockdown, urban density of the area of residence, living in a "priority neighbourhood", quartile of the hospitalisation rate during the first lockdown in the area of residence, body mass index, perceived health status, pre-pandemic chronic mental or physical conditions, tobacco use, and alcohol use

© 2023 Rouquette A et al. *JAMA Network Open.*

eTable 4. Odds Ratio and 95% Confidence Interval of Depression and Anxiety in July 2021 for Patients With a Duration of COVID-19 Symptoms ≤ or > 2 Weeks Before November 2020 Versus Patients Without Symptoms, Crude (OR_c) and Adjusted (OR_a) for Covariates Using Propensity-Score Matching and Conditional Logistic Regression (^{Ps}), and Weighted Logistic Regression (^{Wlr}) in the Entire Sample and Stratified According to SARS-CoV-2 Serology

		N before matching	Number of pairs after matching	Depression			Anxiety		
				OR_c [95% CI]	OR_a [95% CI] ^{ps}	OR_a [95% CI] ^{wlr}	OR_c [95% CI]	OR_a [95% CI] ^{ps}	OR_a [95% CI] ^{wlr}
All		**45 260**							
≤ 2 weeks	No symptom	39 726	3643	1 (Reference)	1 (Reference)	1 (Reference)	1 (Reference)	1 (Reference)	1 (Reference)
	Yes	3646		2.00 [1.80-2.23]	1.52 [1.20-1.91]	1.68 [1.45-1.94]	1.65 [1.44-1.89]	1.27 [1.02-1.57]	1.37 [1.15-1.63]
> 2 weeks	No symptom	39 726	1886	1 (Reference)	1 (Reference)	1 (Reference)	1 (Reference)	1 (Reference)	1 (Reference)
	Yes	1888		2.47 [2.15-2.81]	1.94 [1.48-2.55]	2.02 [1.67-2.43]	2.42 [2.06-2.82]	1.91 [1.27-2.88]	1.79 [1.45-2.20]
Positive SARS-CoV-2 serology		**4348**							
≤ 2 weeks	No symptom	2521	1057	1 (Reference)	1 (Reference)	1 (Reference)	1 (Reference)	1 (Reference)	1 (Reference)
	Yes	1063		1.52 [1.17-1.95]	1.24 [0.90-1.70]	1.29 [0.89-1.87]	1.13 [0.81-1.57]	0.96 [0.61-1.52]	1.04 [0.68-1.59]
> 2 weeks	No symptom	2521	760	1 (Reference)	1 (Reference)	1 (Reference)	1 (Reference)	1 (Reference)	1 (Reference)
	Yes	764		1.87 [1.42-2.44]	1.47 [0.93-2.34]	1.54 [1.08-2.19]	2.13 [1.56-2.89]	1.92 [1.14-3.22]	1.99 [1.36-2.93]
Negative SARS-CoV-2 serology		**40 912**							
≤ 2 weeks	No symptom	37 205	2582	1 (Reference)	1 (Reference)	1 (Reference)	1 (Reference)	1 (Reference)	1 (Reference)
	Yes	2583		2.19 [1.94-2.47]	1.60 [1.19-2.16]	1.81 [1.53-2.14]	1.88 [1.61-2.17]	1.39 [1.01-1.91]	1.52 [1.25-1.85]
> 2 weeks	No symptom	37 205	1121	1 (Reference)	1 (Reference)	1 (Reference)	1 (Reference)	1 (Reference)	1 (Reference)
	Yes	1124		2.84 [2.41-3.34]	2.08 [1.36-3.19]	2.18 [1.72-2.76]	2.62 [2.15-3.16]	2.01 [1.32-3.04]	1.70 [1.31-2.20]

*Covariates used to compute propensity score and as adjustment covariates in weighted logistic regressions were: gender, age, immigration status, highest educational degree, main occupational status, deciles of household income per consumption unit, perceived financial situation, usual residence overcrowded, household structure, living in the usual residence during the first lockdown, access to a private exterior during the first lockdown, urban density of the area of residence, living in a "priority neighbourhood", quartile of the hospitalisation rate during the first lockdown in the area of residence, body mass index, perceived health status, pre-pandemic chronic mental or physical conditions, tobacco use, and alcohol use

© 2023 Rouquette A et al. *JAMA Network Open.*

eTable 5. Crude Odds Ratio (OR_c) and 95% Confidence Interval (95%CI) of Depression and Anxiety in July 2021 for Patients With Positive SARS-CoV-2 Serology Versus Patients With Negative Serology Stratified According to the Duration and Time of Occurrence of COVID-19 Symptoms Before November 2020

		N	Depression	Anxiety
			OR_c [95% CI]	OR_c [95% CI]
Without COVID-19 symptoms		**39 726**		
SARS-CoV2 serology	Negative	37 705	1 (Reference)	1 (Reference)
	Positive	2521	1.04 [0.87-1.20]	1.00 [0.82-1.21]
COVID-19 symptoms > 2 weeks		**1888**		
SARS-CoV2 serology	Negative	939	1 (Reference)	1 (Reference)
	Positive	764	0.69 [0.65-1.11]	0.81 [0.67-1.24]
COVID-19 symptoms ≤ 2 weeks		**3646**		
SARS-CoV2 serology	Negative	2583	1 (Reference)	1 (Reference)
	Positive	1063	0.72 [0.69-1.09]	0.60 [0.59-1.09]
Time of occurrence ≤ 6 months		**2406**		
SARS-CoV2 serology	Negative	1459	1 (Reference)	1 (Reference)
	Positive	947	0.67 [0.66-1.07]	0.72 [0.65-1.16]
Time of occurrence > 6 months		**3128**		
SARS-CoV2 serology	Negative	2248	1 (Reference)	1 (Reference)
	Positive	880	0.73 [0.67-1.13]	0.69 [0.62-1.17]

© 2023 Rouquette A et al. *JAMA Network Open.*

eFigure 1. The EpiCoV Cohort Study Timeline and Data Used in the Present Study

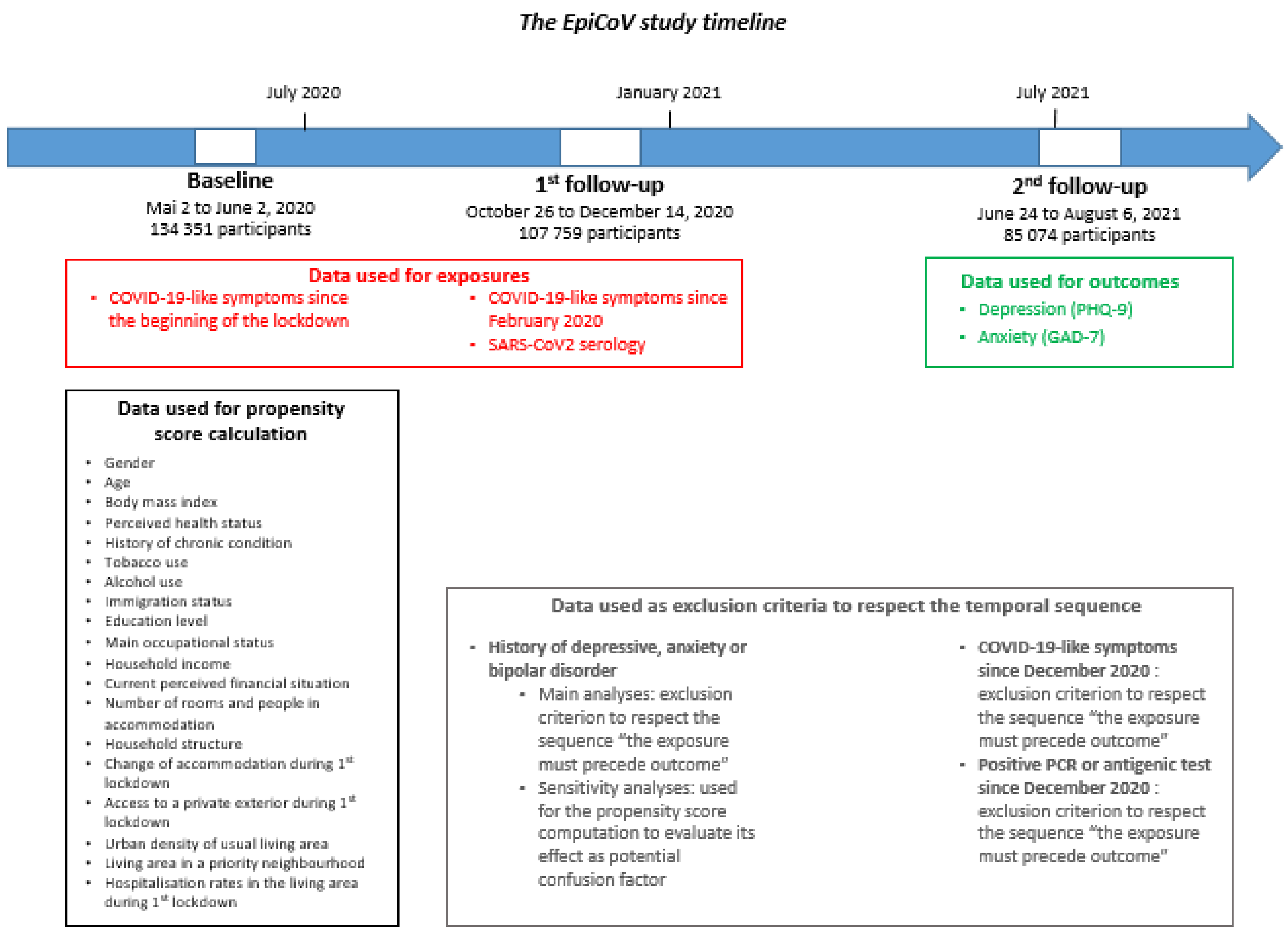

Legend: PHQ-9: Patient Health Questionnaire – 9 items, GAD-7: Generalized Anxiety Disorder – 7 items.

© 2023 Rouquette A et al. *JAMA Network Open.*

Labels of the variables used in the following eFigures 2 to 20 that shows the covariates balance before and after matching in propensity score analyses

1: Gender
2: Age
3: Immigration status
  3_0: Participant and parents born in mainland France
  3_1: Participant and/or parents born in overseas territories
  3_2: Native from France, parents born outside France
  3_3: Born outside France
4: Highest educational diploma
  4_0: None
  4_1: Lower secondary school certificate
  4_2: Professional certificate
  4_3: Higher secondary school certificate
  4_4: Bachelor degree or equivalent
  Master degree or more
5: Main occupational status before the first lockdown
  5_0: Employed
  5_1: Students
  5_2: Unemployed
  5_3: Retired
  5_4: Other situations
6: Household income per consumption units
  6_0: First and second decile (poorest households)
  6_1: Third and fourth deciles
  6_2: Fifth and sixth deciles (median ones)
  6_3: Seventh and eighth deciles
  6_4: Ninth and tenth deciles (wealthiest households)
7: Perceived financial situation
  7_3: Comfortable
  7_2: Decent
  7_1: Short
  7_0: Difficult or unbearable without making debt
8: Overcrowded usual housing
9: Household structure
  9_0: Single
  9_1: Couple without children
  9_2: Couple with children
  9_3: Single-parent family
  9_4: Child living at family house
  9_5: Complex household
10: Usual housing during the first lockdown

11: Housing with private exterior during the first lockdown
12: Urban density of living area (urban units)
  12_0: Oversea territories
  12_1: Rural
  12_2: Between 2000 and 1 999 999
  12_3: Paris area
13: Priority neighbourhood
14: Body mass index (kg/m²)
  14_0: <18·5
  14_1: ≥18·5 and <25
  14_2: ≥25 and <30
  14_3: ≥ 30
15: Perceived health status
  15_2: Well to very well
  15_1: Quite well
  15_0: Poor to very poor
16: Pre-pandemic chronic somatic condition
17: Pre-pandemic chronic psychiatric condition (other than mood disorders)
18: Tobacco use
  18_0: Never
  18_1: Former
  18_2: Current
19: Alcohol use
  19_0: Never
  19_1: Rare
  19_2: Occasional
  19_3: Often
  19_4: Daily
20: Quartile of hospitalization rates in 1st LD department
  20_0: Q1
  20_1: Q2
  20_2: Q3
  20_3: Q4
21: Usual living area by intensity of the first Covid19 wave
  21_0: Low
  21_1: Moderate
  21_2: High
  21_3: Very high

© 2023 Rouquette A et al. *JAMA Network Open.*

eFigure 2. Balance in Propensity Score Matching: Absolute Mean Difference (Circle) and Range (Line) Across Imputations for Participants With or Without **COVID-19 Symptoms**, Before (Unadjusted) and After Matching (Adjusted, N=5531 Pairs) Performed Within Each 5 Imputed Datasets

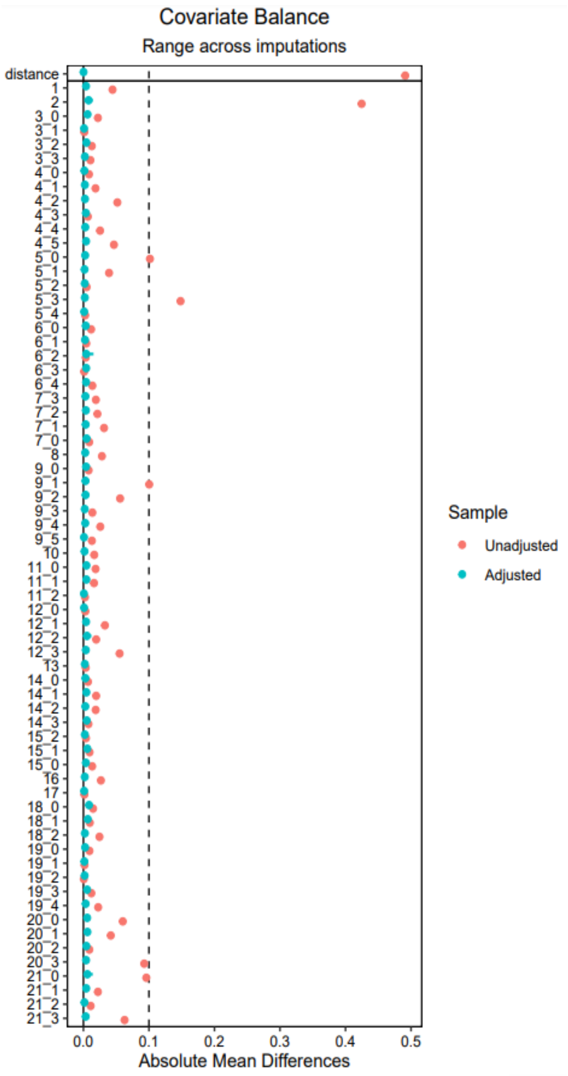

eFigure 3. Balance in Propensity Score Matching: Absolute Mean Difference (Circle) and Range (Line) Across Imputations for Participants With or Without **COVID-19 Symptoms, in the Positive SARS-CoV-2** Serology Subsample, Before (Unadjusted) and After Matching (Adjusted, N=1806 Pairs) Performed Within Each 5 Imputed Datasets

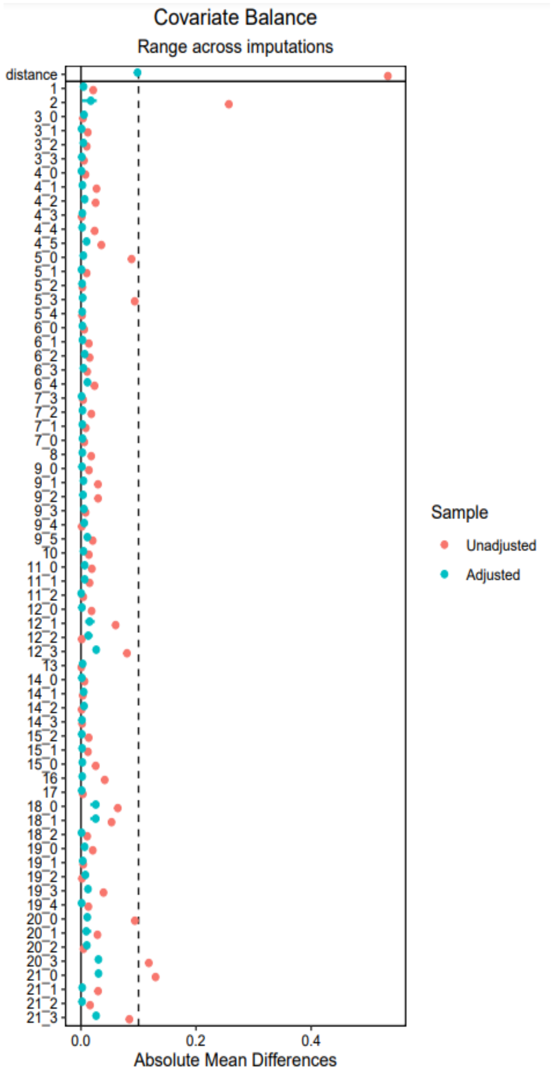

eFigure 4. Balance in Propensity Score Matching: Absolute Mean Difference (Circle) and Range (Line) Across Imputations for Participants With or Without **COVID-19 Symptoms, in the Negative SARS-CoV-2** Serology Subsample, Before (Unadjusted) and After Matching (Adjusted, N=3706 Pairs) Performed Within Each 5 Imputed Datasets

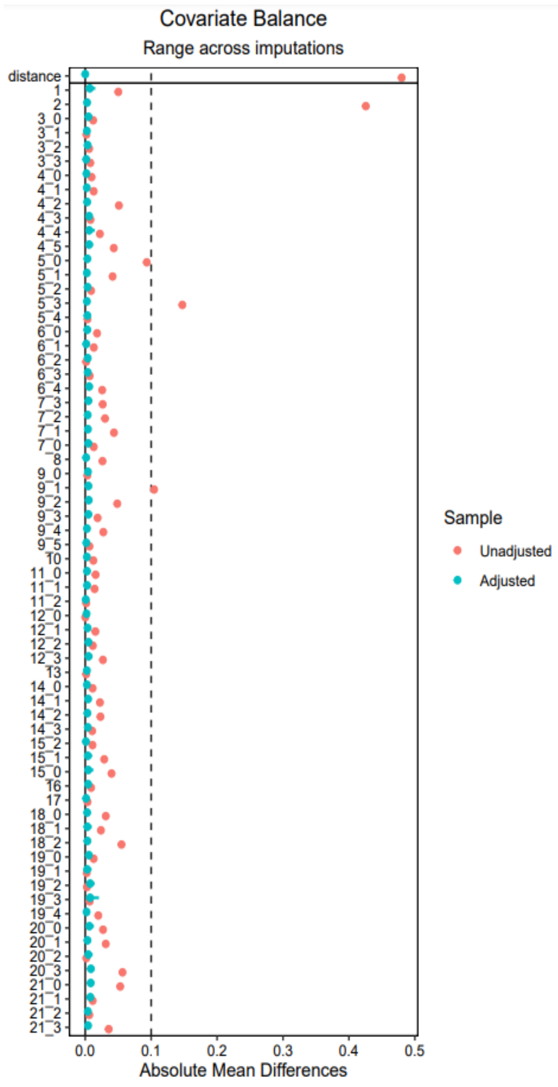

© 2023 Rouquette A et al. *JAMA Network Open.*

eFigure 5. Balance in Propensity Score Matching: Absolute Mean Difference (Circle) and Range (Line) Across Imputations for Participants With or Without **Anosmia and/or Dysgeusia**, Before (Unadjusted) and After Matching (Adjusted, N=2355 Pairs) Performed Within Each 5 Imputed Datasets

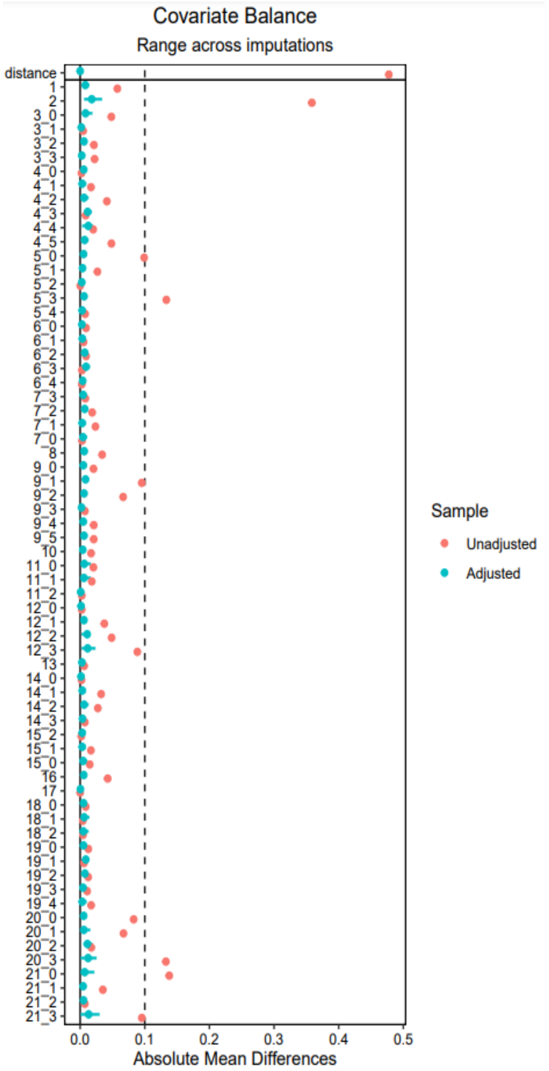

eFigure 6. Balance in Propensity Score Matching: Absolute Mean Difference (Circle) and Range (Line) Across Imputations for Participants With or Without **Anosmia and/or Dysgeusia, in the Positive SARS-CoV-2** Serology Subsample, Before (Unadjusted) and After Matching (Adjusted, N=1323 Pairs) Performed Within Each 5 Imputed Datasets

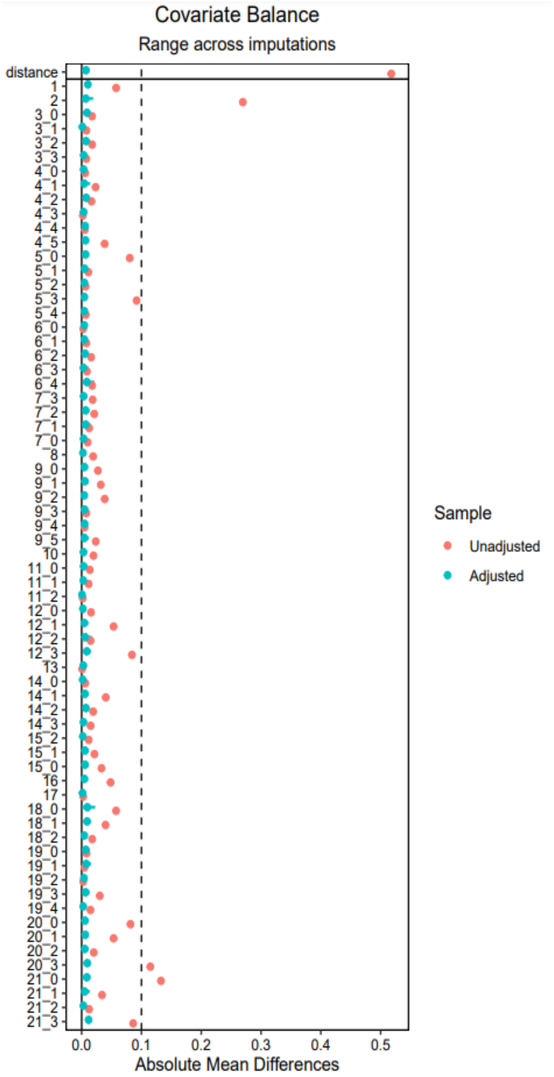

eFigure 7. Balance in Propensity Score Matching: Absolute Mean Difference (Circle) and Range (Line) Across Imputations for Participants With or Without **Anosmia and/or Dysgeusia, in the Negative SARS-CoV-2** Serology Subsample, Before (Unadjusted) and After Matching (Adjusted, N=1032 Pairs) Performed Within Each 5 Imputed Datasets

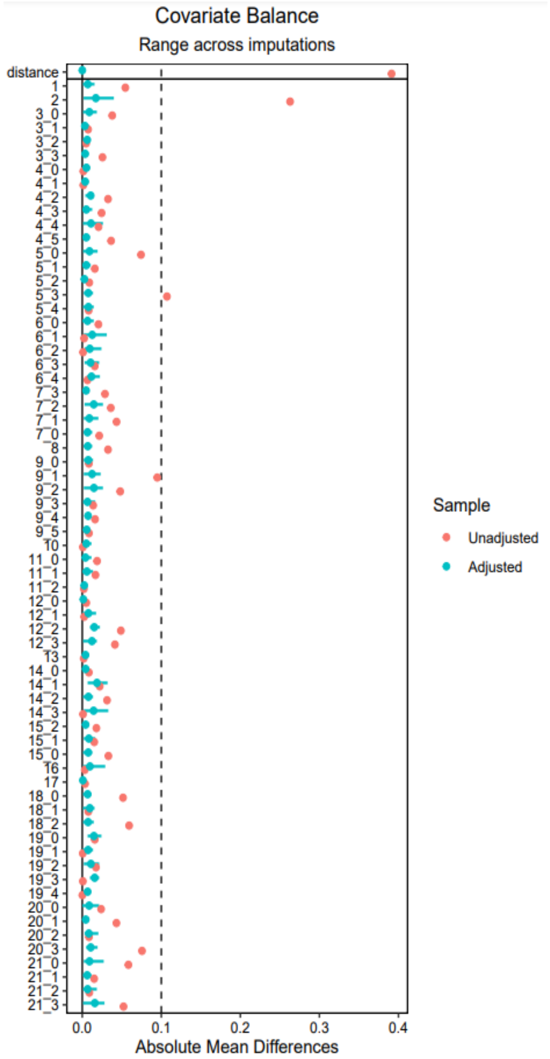

© 2023 Rouquette A et al. *JAMA Network Open.*

eFigure 8. Balance in Propensity Score Matching: Absolute Mean Difference (Circle) and Range (Line) Across Imputations for Participants With or Without **SARS-CoV-2 Serology**, Before (Unadjusted) and After Matching (Adjusted, N=4348 Pairs) Performed Within Each 5 Imputed Datasets

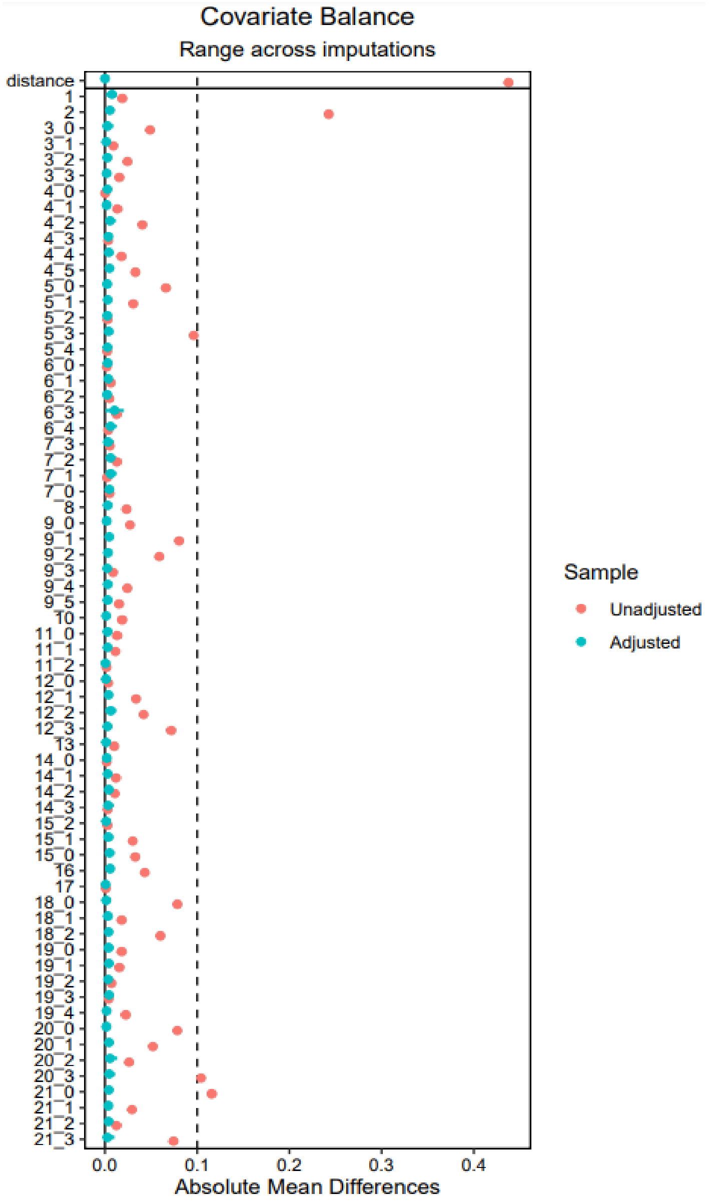

© 2023 Rouquette A et al. *JAMA Network Open.*

eFigure 9. Balance in Propensity Score Matching: Absolute Mean Difference (Circle) and Range (Line) Across Imputations for Participants With **History of COVID-19 Symptoms ≤ 6 Months or No Symptom**, Before (Unadjusted) and After Matching (Adjusted, N=2405 Pairs) Performed Within Each 5 Imputed Datasets

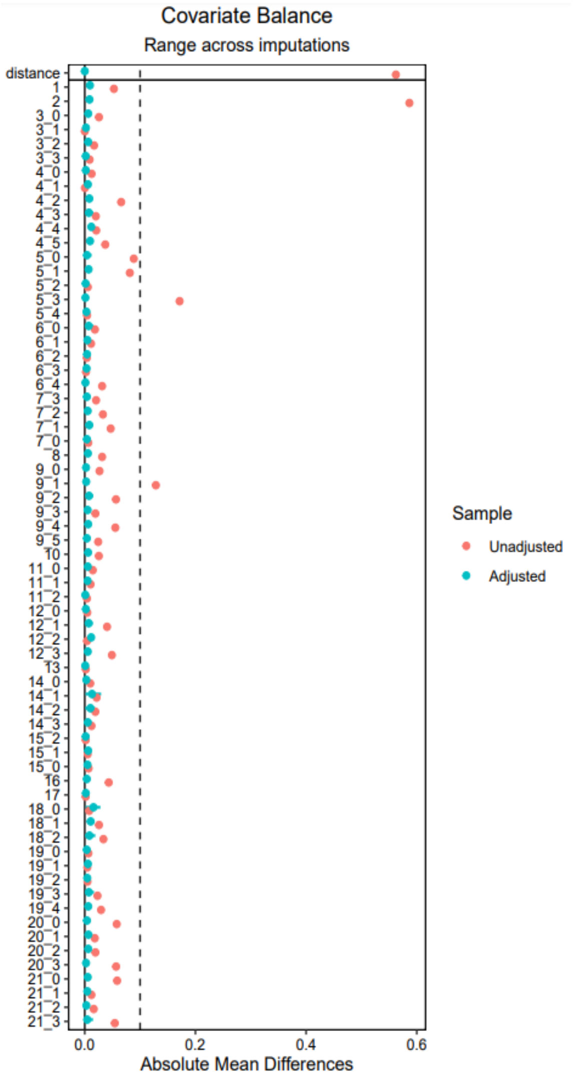

eFigure 10. Balance in Propensity Score Matching: Absolute Mean Difference (Circle) and Range (Line) Across Imputations for Participants With **History of COVID-19 Symptoms ≤ 6 Months Or No Symptom, in the Positive SARS-CoV-2** Serology Subsample, Before (Unadjusted) and After Matching (Adjusted, N=937 Pairs) Performed Within Each 5 Imputed Datasets

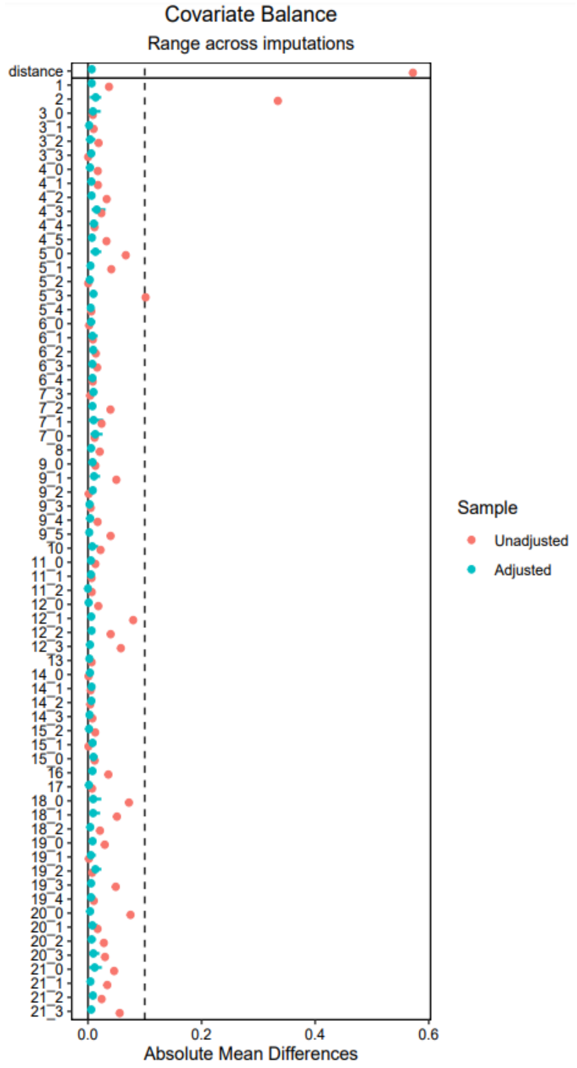

eFigure 11. Balance in Propensity Score Matching: Absolute Mean Difference (Circle) and Range (Line) Across Imputations for Participants With **History of COVID-19 Symptoms ≤ 6 Months or No Symptom, in the Negative SARS-CoV-2** Serology Subsample, Before (Unadjusted) and After Matching (Adjusted, N=1459 Pairs) Performed Within Each 5 Imputed Datasets

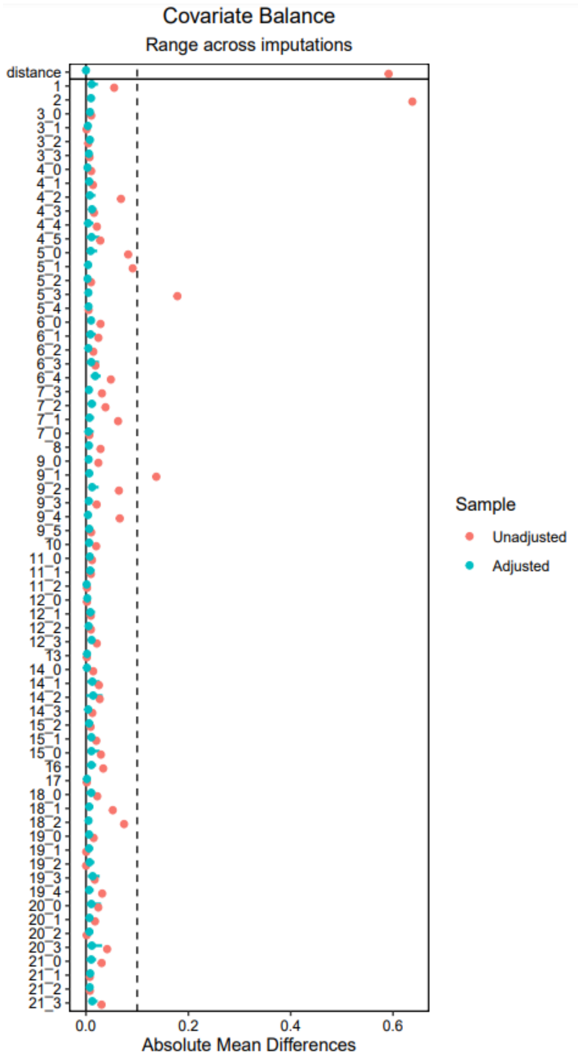

© 2023 Rouquette A et al. *JAMA Network Open.*

eFigure 12. Balance in Propensity Score Matching: Absolute Mean Difference (Circle) and Range (Line) Across Imputations for Participants With **History of COVID-19 Symptoms > 6 Months or No Symptom**, Before (Unadjusted) and After Matching (Adjusted, N=3127 Pairs) Performed Within Each 5 Imputed Datasets

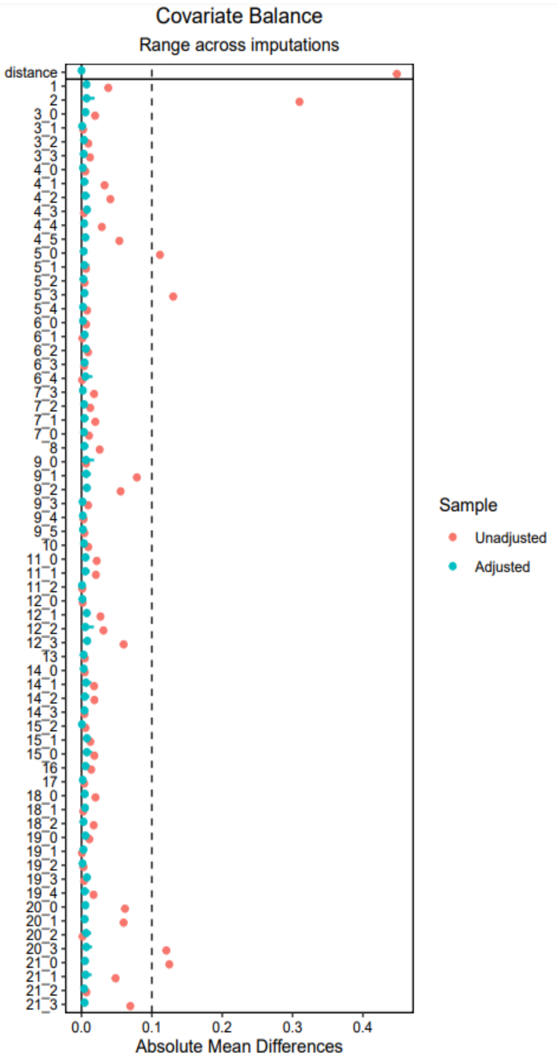

eFigure 13. Balance in Propensity Score Matching: Absolute Mean Difference (Circle) and Range (Line) Across Imputations for Participants With **History of COVID-19 Symptoms > 6 Months or No Symptom, in the Positive SARS-CoV-2** Serology Subsample, Before (Unadjusted) and After Matching (Adjusted, N=879 Pairs) Performed Within Each 5 Imputed Datasets

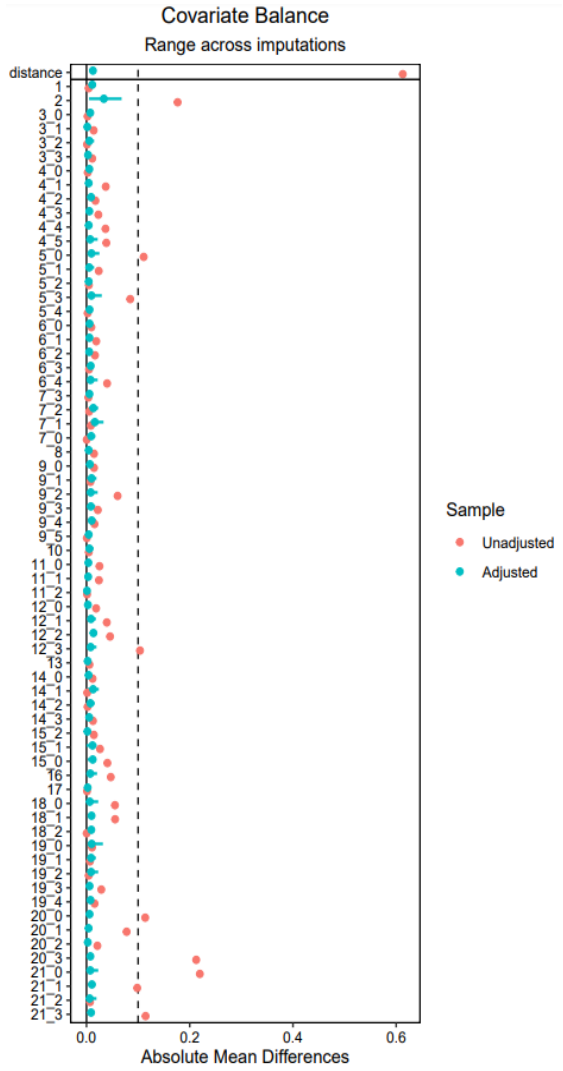

eFigure 14. Balance in Propensity Score Matching: Absolute Mean Difference (Circle) and Range (Line) Across Imputations for Participants With **History of COVID-19 Symptoms > 6 Months or No Symptom, in the Negative SARS-Cov-2** Serology Subsample, Before (Unadjusted) and After Matching (Adjusted, N=2247 Pairs) Performed Within Each 5 Imputed Datasets

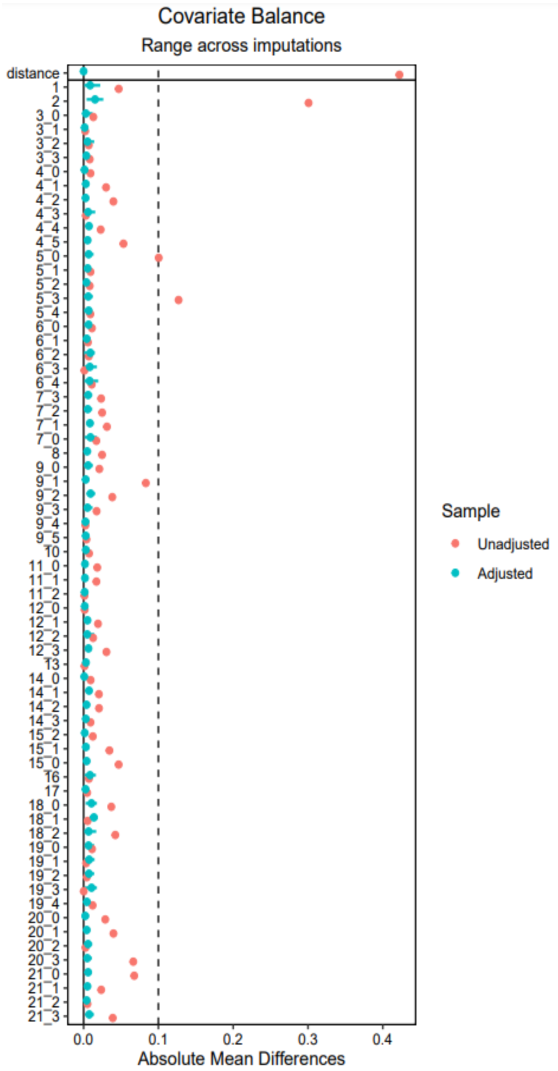

© 2023 Rouquette A et al. *JAMA Network Open.*

eFigure 15. Balance in Propensity Score Matching: Absolute Mean Difference (Circle) and Range (Line) Across Imputations for Participants With **Duration of COVID-19 Symptoms ≤ 2 Weeks or No Symptom**, Before (Unadjusted) and After Matching (Adjusted, N=3643 Pairs) Performed Within Each 5 Imputed Datasets

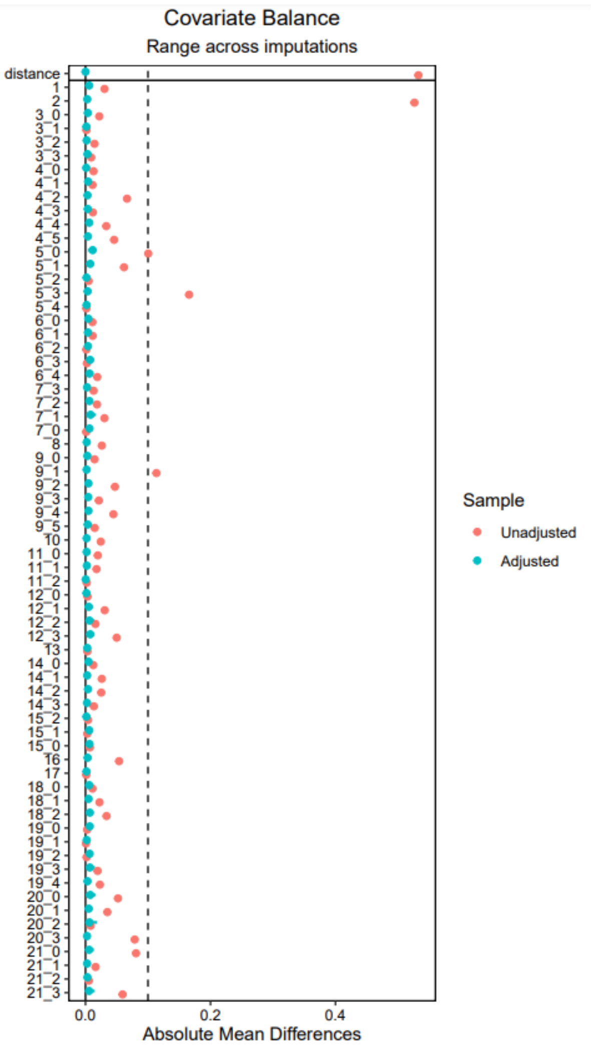

eFigure 16. Balance in Propensity Score Matching: Absolute Mean Difference (Circle) and Range (Line) Across Imputations for Participants With **Duration of COVID-19 Symptoms ≤ 2 Weeks or No Symptom, in the Positive SARS-CoV-2** Serology Subsample, Before (Unadjusted) and After Matching (Adjusted, N=1057 Pairs) Performed Within Each 5 Imputed Datasets

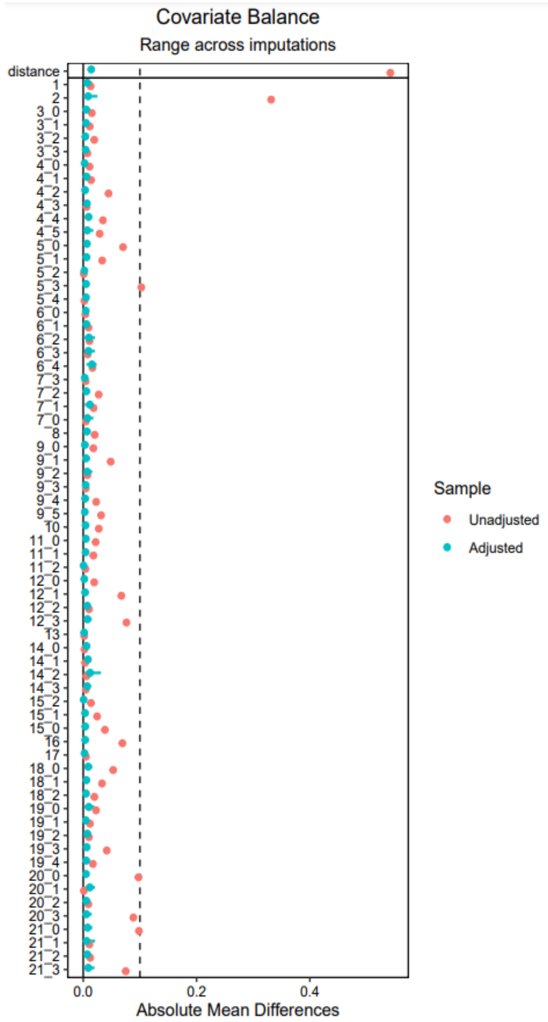

eFigure 17. Balance in Propensity Score Matching: Absolute Mean Difference (Circle) and Range (Line) Across Imputations for Participants With **Duration of COVID-19 Symptoms ≤ 2 Weeks or No Symptom, in the Negative SARS-CoV-2** Serology Subsample, Before (Unadjusted) and After Matching (Adjusted, N=2582 Pairs) Performed Within Each 5 Imputed Datasets

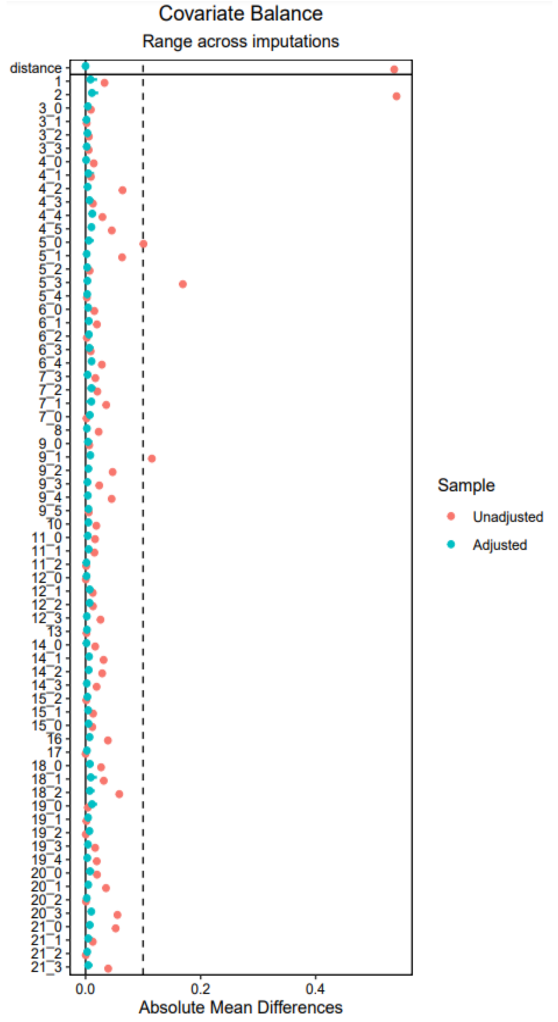

© 2023 Rouquette A et al. *JAMA Network Open.*

eFigure 18. Balance in Propensity Score Matching: Absolute Mean Difference (Circle) and Range (Line) Across Imputations for Participants With **Duration of COVID-19 Symptoms > 2 Weeks or No Symptom**, Before (Unadjusted) and After Matching (Adjusted, N=1886 Pairs) Performed Within Each 5 Imputed Datasets

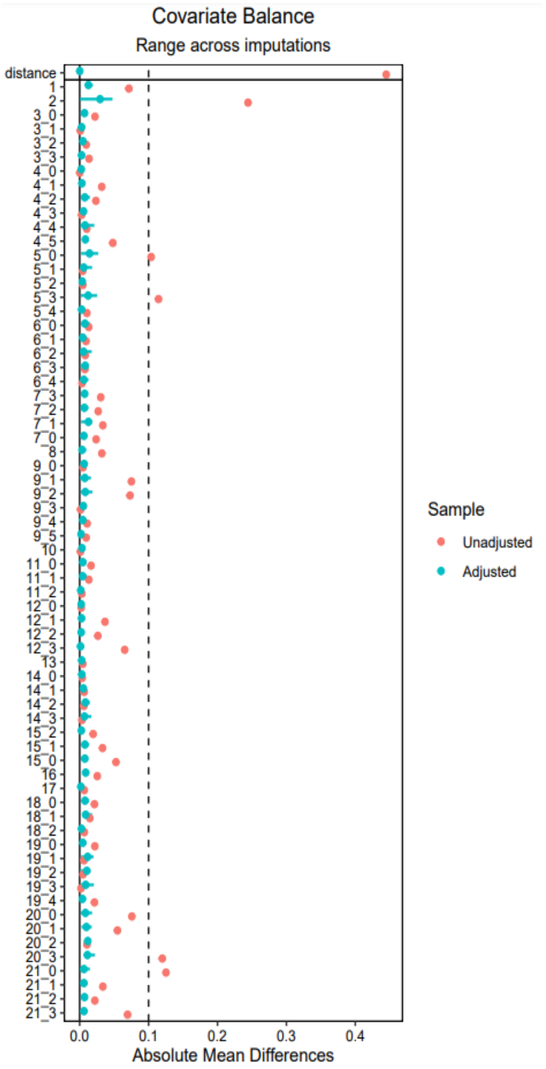

eFigure 19. Balance in Propensity Score Matching: Absolute Mean Difference (Circle) and Range (Line) Across Imputations for Participants With **Duration of COVID-19 Symptoms > 2 Weeks or No Symptom, in the Positive SARS-CoV-2** Serology Subsample, Before (Unadjusted) and After Matching (Adjusted, N=760 Pairs) Performed Within Each 5 Imputed Datasets

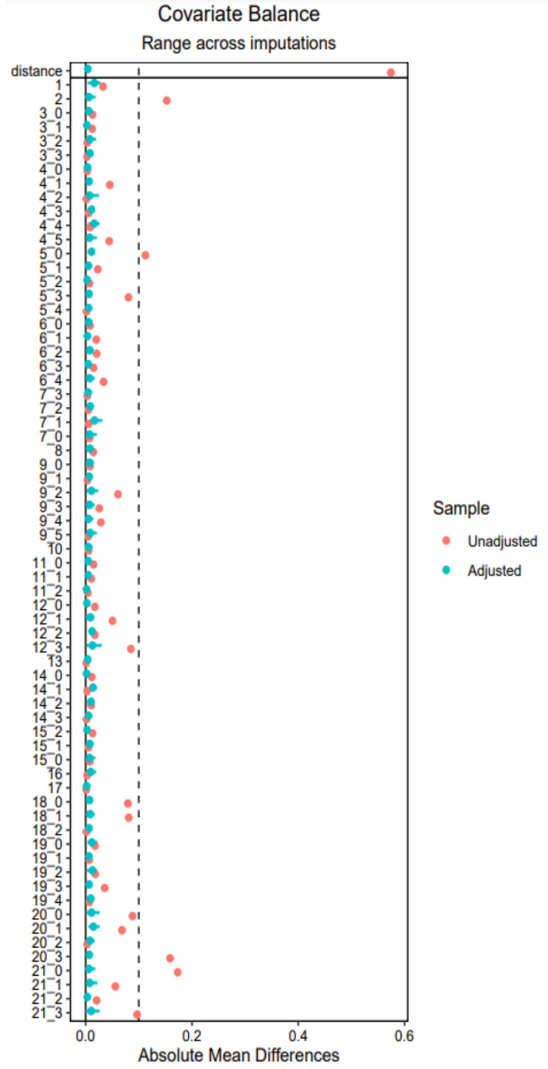

eFigure 20. Balance in Propensity Score Matching: Absolute Mean Difference (Circle) and Range (Line) Across Imputations For Participants With **Duration of COVID-19 Symptoms > 2 Weeks or No Symptom, in the Negative SARS-CoV-2** Serology Subsample, Before (Unadjusted) and After Matching (Adjusted, N=1121 Pairs) Performed Within Each 5 Imputed Datasets

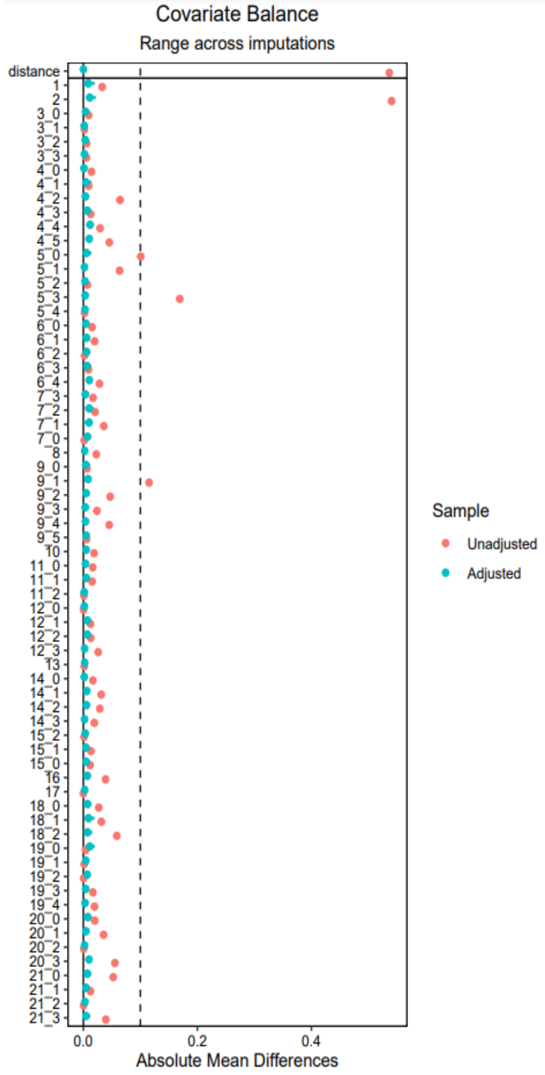

© 2023 Rouquette A et al. *JAMA Network Open.*
