# Supplementary material for: Comparison of Depression and Anxiety Following Self-reported COVID-19–Like Symptoms vs SARS-CoV-2 Seropositivity in France
Source: JAMA Netw Open. 2023 May 11;6(5):e2312892. doi: 10.1001/jamanetworkopen.2023.12892 (PMC10176124; doi:10.1001/jamanetworkopen.2023.12892)
Supplement: Supplement 2. — Nonauthor Collaborators [file jamanetwopen-e2312892-s002.pdf]

Supplemental Online Content: Nonauthor Collaborators
*First name, last name, and suffix (if applicable) are required and will appear in PubMed.

***Group Name(s): EpiCoV Study Group**							
***First Name and Middle Initial(s)**	***Last Name**	***Suffix (eg, Jr, III)**	Academic Degrees	Institution	Location (city, state/province, country)	Role or Contribution, eg, chair, principal investigator	Group (if more than 1 Group listed in the byline) and/or Subgroup (eg, Steering Committee)
Bajos	Nathalie		PhD	IRIS, Inserm/EHESS/CNRS	Aubervilliers, France	Co-principal Investigator	
Bagein	Guillaume		MSc	DREES-Direction de la Recherche, des Etudes, de l'évaluation et des statistiques	Paris, France		
Beck	François		PhD	Santé Publique France	Saint-Maurice, France		
Counil	Emilie		PhD	1 - IRIS, Inserm/EHESS/CNRS<br>2 - Ined	Aubervilliers, France		
Jusot	Florence		PhD	Université Paris Dauphine	Paris, France		
Lydie	Nathalie		PhD	Santé Publique France	Saint-Maurice, France		
Martin	Claude		PhD	ARENES UMR 6051, CNRS, EHESP	Rennes, France		
Raynaud	Philippe		PhD	DREES-Direction de la Recherche, des Etudes, de l'évaluation et des statistiques	Paris, France		
Pailhe	Ariane		PhD	Ined	Aubervilliers, France		
Rahib	Delphine		PhD	Santé Publique France	Saint-Maurice, France		
Sillard	Patrick		PhD	Institut National de la statistique et des études économiques	Montrouge, France		
Slama	Rémy		PhD	1 - Institut thématique de Santé Publique, INSERM<br>2 - Inserm, CNRS, Team of Environmental Epidemiology applied to Reproduction and Respiratory Health, Institute for Advanced Biosciences, University Grenoble Alpes	1 - Paris, France<br>2 - Grenoble, France		
Spire	Alexis		PhD	IRIS, Inserm/EHESS/CNRS	Aubervilliers, France		
