# Supplementary material for: Comparison of Depression and Anxiety Following Self-reported COVID-19–Like Symptoms vs SARS-CoV-2 Seropositivity in France
Source: JAMA Netw Open. 2023 May 11;6(5):e2312892. doi: 10.1001/jamanetworkopen.2023.12892 (PMC10176124; doi:10.1001/jamanetworkopen.2023.12892)
Supplement: Supplement 3. — Data Sharing Statement [file jamanetwopen-e2312892-s003.pdf]

Data Sharing Statement

Rouquette. Comparison of Depression and Anxiety Following Self-reported COVID-19-Like Symptoms vs SARS-CoV-2 Seropositivity in France. *JAMA Netw Open*. Published May 11, 2023. doi:10.1001/jamanetworkopen.2023.12892

Data
Data available: Yes
Data types: Deidentified participant data, Data dictionary
How to access data: The EpiCov dataset is available for research purposes concerning the baseline and first and second follow-ups on CASD (https://www.casd.eu/)
When available: With publication

Supporting Documents
Document types: None

Additional Information
Who can access the data: Any researcher after submission to the EpiCoV data operation committee (mail to the corresponding author) for approval according to the French Ethics and Regulatory Committee procedure (Comité du Secret Statistique, CESREES and CNIL).
Types of analyses: for research purposes
Mechanisms of data availability: with a signed data access agreement
